# Usability and Usefulness of Machine Learning–Based Clinical Decision Support Software in Primary Care: Survey of Users in a Prospective Observational Study

**DOI:** 10.2196/80527

**Published:** 2026-05-27

**Authors:** Willem Ernst Herter, Janine Khuc, Tobias N Bonten, Robert A Verheij, Mattijs E Numans, Niels H Chavannes

**Affiliations:** 1 Department of Public Health and Primary Care, Leiden University Medical Center, Albinusdreef 2, Leiden, 2333 ZA, The Netherlands, 31 629292797; 2 Pacmed, Amsterdam, The Netherlands; 3 Netherlands Institute for Health Services Research, Utrecht, The Netherlands; 4 Tilburg School of Social and Behavioral Sciences, Tilburg University, Tilburg, The Netherlands; 5 National Health Care Institute, Diemen, The Netherlands

**Keywords:** machine learning, artificial intelligence, clinical decision support system, implementation study, surveys and questionnaires

## Abstract

**Background:**

The successful implementation of decision support systems promises to enhance high-quality care. However, the successful implementation of a clinical decision support system (CDSS) depends on user acceptance and adoption. A machine learning (ML)–based CDSS to assist primary care professionals treating urinary tract infections (UTIs) was implemented, and usability and usefulness were assessed through a questionnaire.

**Objective:**

This study aimed to assess the system’s usability by examining users’ experiences with the software. A secondary goal was to assess users’ attitudes toward evidence-based practice and innovation in health care.

**Methods:**

In collaboration with the Netherlands Institute for Health Services Research (NIVEL) and Leiden University Medical Center (LUMC), Pacmed Ltd developed the CDSS. The cohort was mostly recruited at the care group level; practices within participating care groups were required to participate. Health insurers partly funded the research. Practitioners participated in the implementation study for 4 months. A survey based on the Unified Theory of Acceptance and Use of Technology (UTAUT) was sent to 263 general practitioners and assistants shortly after the implementation period. Furthermore, usage data were analyzed.

**Results:**

Of the 34 participating practices that used the software, 30 (88%) submitted at least one survey response, with a mean of 2.23 responses per practice (SD 1.43). The CDSS was used throughout the pilot period, and 31 practices continued using the tool, with 9% dropping out during the first 8 weeks. Sixty-seven percent of respondents trusted the tool’s output, and 73% found it understandable how the algorithm came to predictions. Sixty-five percent of respondents indicated that the information provided was useful in addition to the available guidelines, and 52% agreed that it supported their decision-making. However, many respondents were uncertain whether the tool improved patient care (46%) or patient outcomes (66%). Forty-eight percent of respondents found the software easy to integrate into their clinical workflow.

**Conclusions:**

The CDSS was perceived as trustworthy and easy to use. However, users were unable to determine whether the CDSS improved patient outcomes. In addition, the CDSS development could have benefited from including assistants as well as general practitioners more in the design phase of the software. Because assistants play an important role in UTI care, designing the software to better fit existing workflows may reduce the perceived time investment associated with using the tool. Finally, respondents reported strong motivation to contribute to further research in this field and indicated willingness to embrace change in health care delivery, which may also reflect selection bias in our sample.

## Introduction

### Background

Machine learning (ML)–based applications are increasingly being developed for use in health care, and a growing body of this work is published in peer-reviewed journals [1]. However, only a small fraction of these applications are ultimately implemented in clinical practice, and their efficacy and usability in real-world settings are rarely evaluated [2].

The implementation of ML and data analytics in health care faces many challenges. One key barrier is health care professionals’ willingness to adopt clinical decision support systems (CDSS) and to engage with insights derived from electronic health record (EHR) data. To explore these implementation challenges, we studied the introduction of a CDSS designed to support general practitioners (GPs) in selecting appropriate treatments for patients with urinary tract infections (UTIs).

With approximately 70 per 1000 female patients and almost 10 per 1000 male patients, UTI is one of the most common diagnoses in primary care. Effective UTI treatment has a high impact on quality of care and cost-effectiveness [3,4]. At the time of this study, the Dutch Association of General Practitioners (NHG) guideline in the Netherlands was based on 21 randomized controlled trials (RCTs) and underrepresented patients belonging to guideline-defined risk groups. As a result, evidence supporting recommendations for several risk groups was limited. Given the complexity and heterogeneity of UTI presentations in practice, treating all patients efficiently remains challenging. GPs therefore reported lack of agreement as a problem for all key recommendations while using that UTI guideline at the time of our intervention [5].

GPs have a gatekeeping role in the Dutch health care system, controlling patients’ access to specialized care. GPs are usually the first health professionals to consult, and there is no copayment. Virtually all Dutch residents are registered in a GP practice, and nearly all GP practices use EHRs. This creates opportunities to identify determinants of treatment success and failure using routinely collected clinical data. Prior work has shown that reusing this data by mining it can help us learn about treatment effectiveness and outcomes on a real patient population, revealing unknown disease correlations in the process, resulting in increasing quality of care [6-8]. In a collaboration of Netherlands Institute for Health Services Research (NIVEL), Leiden University Medical Center (LUMC), and Pacmed (Ltd), a data-driven CDSS to aid the efficient treatment of UTIs was built. The aim was to support GPs and patients with UTI treatment decisions. The CDSS was implemented in 36 general practices and was associated with a statistically significant improvement in treatment success [9].

### Goal of This Study

CDSSs have the potential to improve the quality of care and patient outcomes by helping health care professionals process large amounts of information. Despite the promise, studies consistently show that the mere provision of CDSSs does not guarantee their uptake [9-11]. Even where CDSSs are available and have demonstrated improved outcomes, they may fall short of their full potential because of limited adoption [4,11-13]. In the Netherlands, approximately 21% of GPs reported having access to a CDSS, and only about one-fifth reported using these during consultations [14,15]. Low adoption rates of decision support systems remain a major obstacle to realizing the full potential of CDSS.

Multiple factors contributing to limited CDSS adoption have been identified. Adoption in health care is often driven by users’ perceptions of usefulness, including the relevance and applicability of information to individual patients, trust in the system, and fit with clinical workflows [16-22]. In addition, the user’s attitudes toward changes in health care and evidence-based practice influence CDSS adoption [21,23]. These factors do not fully explain the limited adoption of CDSS, underscoring the need to better understand barriers to technology adoption among health care professionals. Furthermore, more elaborate research on possible risks and pitfalls of implementing decision support warrants further study. For example, decision support may contribute to overreliance on automated recommendations, potentially reducing care quality if clinicians do not critically appraise the presented information [24,25].

Several of these issues were addressed during the CDSS design phase, with the aim of maximizing applicability, interpretability, usability, and trust. To evaluate the usability and perceived usefulness of the CDSS, an implementation study was conducted in 36 primary care practices for a period of 4 months. The goal of this paper is to evaluate the usability and perceived usefulness by examining the user’s use and experience with the software.

## Methods

### Recruitment

This was a quantitative interventional design. The cohort included 36 practices and 263 users in total (116 GPs and 147 assistants). The cohort was mostly recruited on the care group level, and all the practices within a care group were required to participate. In the Netherlands, care groups are collaborative organizations of health care providers that jointly coordinate specific elements of care delivery, such as chronic care management or participation in innovation and quality improvement programs. Practices participated in the implementation study for a period of 4 months. A 4-month implementation period was chosen based on a power analysis for the primary clinical outcome (change in treatment success for patients with UTI), as reported in our companion paper [9]. Clinicians at participating practices were trained in responsible use of the software and were instructed to use it to support decision-making for patients presenting with a UTI (ClinicalTrials.gov NCT04408976). As part of the study, a survey was conducted among all users to evaluate the usability and perceived usefulness of the software. Use of the software was nonmandatory, and no financial or other incentives were associated with its use.

This usability and perceived usefulness study was cross-sectional and used an online survey sent to all 263 users after the intervention period in 2018.

Use of the CDSS was recorded within the tool. Clinical outcomes and changes in prescribing behavior during the implementation period are reported in a separate paper [9]. This study evaluates usability, perceived usefulness, and acceptance of the CDSS after real-world use. Accordingly, we did not include a parallel control group because respondents needed to have interacted with the CDSS to meaningfully assess usability and perceived value.

### Pacmed’s CDSS

**Figure 1.**
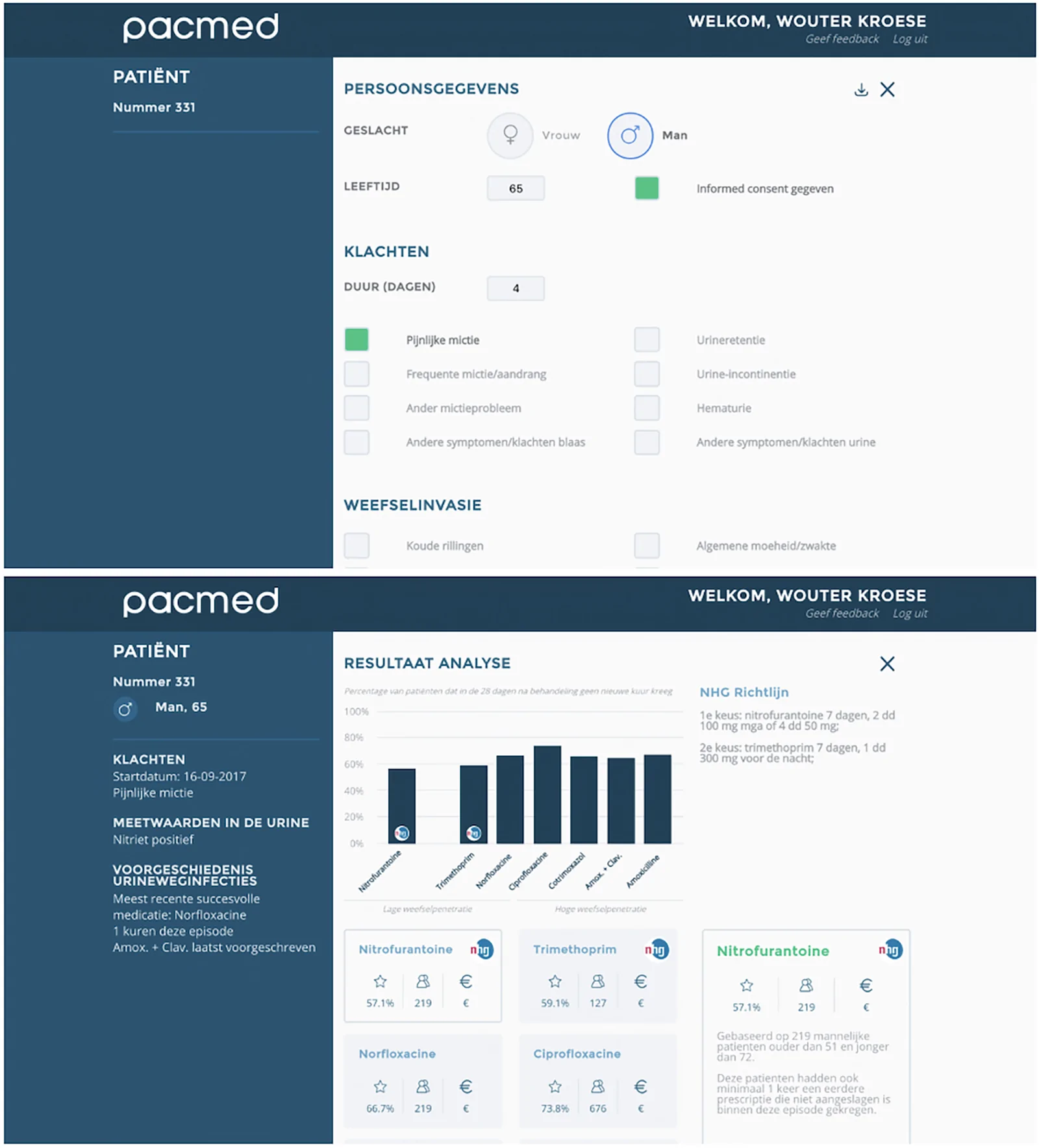
Decision support software: interface to enter patient characteristics (top); presentation of expected outcomes and NHG (Dutch College of General Practitioners) guidelines (bottom).

The decision support software was developed as a collaborative effort between NIVEL, LUMC, and Pacmed. The software uses algorithms based on the analysis of data from the NIVEL Primary Care Database, which included a total of 122,203 UTIs [26]. The software interface was developed in close collaboration with several primary care physicians through user tests and expert groups. Users were requested to enter patients’ characteristics, and the CDSS then provided the users with a bar chart of the expected outcomes for the relevant treatment options based on similar patients in the database. In addition, the relevant part of the NHG guideline was displayed. Additional information supporting the expected outcomes could be retrieved by clicking on a treatment option. This information included the number of patients the analysis was based on, in addition to an age band of these patients and other clinically relevant features (Figure 1). Further details on the ML methods and datasets used for model training and validation are reported in our companion paper [9]. Before implementation, software use was explained in training sessions. During the implementation period, the interactions with the users were minimal and occurred primarily in response to feedback or support requests.

### Usage Data

To investigate usage behavior with Pacmed’s clinical decision support software, data of the user’s interaction with the software was analyzed. Usage behavior was continuously recorded as users interacted with the software, which includes the time of service and the number of logins, as well as the activities mentioned above (creating new patient files, editing patient files, accessing patient files, medications suggested before and after results presentation, and provision of feedback to the developers).

### Questionnaire

The questionnaire was developed using the unified theory of technology acceptance model as a structural model that was designed to measure users’ experiences with the software and several of the health care professionals’ attitudes and characteristics [27]. Of the 41 questions, 28 were Likert scale-based questions, where respondents were requested to indicate their level of agreement on a 5-point Likert scale (1: strongly disagree-5: strongly agree), with an additional “not applicable” response option.

Twenty of the 28 questions represented key operationalizations from the unified theory of acceptance and use of technology (UTAUT; concepts included ease of use, usefulness, trust in the system, and facilitating conditions [20,28,29]). An additional 4 questions were added to explore the system quality and satisfaction, and 4 more questions were added to explore users’ intention to increase evidence-based practices in their daily activities, their opinion about the Dutch College of General Practitioners (DCGP or NHG), and their opinion toward the use of observational data for medical research [29]. Four open-ended questions were asked to allow more elaborate responses. These questions requested responses regarding how the software fit into their workflow, their personal most important reason to use the software, suggestions to improve information quality, and suggestions for improvement to the software. Finally, demographic and professional information were also requested (age, gender, years of experience, and previous use of CDSS).

To ensure the content validity of the questions, the draft questionnaire was reviewed by 4 GPs that did not participate in the implementation study but are familiar with the type of decision support that was being evaluated. The quality and reliability of the individual items were evaluated and improved using the Survey Quality Predictor (SQP). SQP is an online tool with an open-source database of questionnaire items and quality estimates that allows evaluation and improvement of survey questions during instrument development [30,31]. The question characteristics were coded by 2 authors (WEH and JK). The questions were improved on the basis of the quality prediction of SQP, as well as information about the effects of individual characteristics of the questions on the quality and validity. The reliability, validity, and quality coefficient estimates can be found in Table 1, and the accompanying statements in Table 2.

**Table 1.** Predicted survey quality predictor (SQP) reliability, validity, and quality coefficients (0‐1), including SEs and method effect for each question.

Question	Reliability coefficients	SE (reliability)	Validity coefficients	SE (validity)	Quality coefficients	SE (quality)	Method effect
Q1	0.85	0.15	0.97	0.13	0.82	0.11	0.24
Q2	0.84	0.14	0.97	0.12	0.81	0.10	0.23
Q6	0.85	0.15	0.97	0.13	0.83	0.11	0.24
Q7	0.83	0.14	0.97	0.12	0.81	0.10	0.24
Q8	0.84	0.14	0.97	0.12	0.81	0.10	0.23
Q9	0.81	0.15	0.96	0.12	0.78	0.11	0.27
Q10	0.83	0.13	0.97	0.13	0.80	0.10	0.23
Q13	0.86	0.15	0.97	0.12	0.84	0.11	0.23
Q14	0.82	0.15	0.97	0.12	0.79	0.11	0.26
Q15	0.81	0.15	0.97	0.13	0.78	0.11	0.26
Q16	0.83	0.15	0.96	0.15	0.80	0.12	0.28
Q18	0.83	0.16	0.96	0.14	0.80	0.12	0.29
Q19	0.82	0.13	0.97	0.13	0.80	0.10	0.26
Q20	0.81	0.16	0.95	0.16	0.77	0.12	0.31
Q21	0.81	0.15	0.96	0.12	0.78	0.11	0.29
Q22	0.83	0.14	0.97	0.13	0.81	0.10	0.25
Q23	0.83	0.14	0.96	0.14	0.80	0.11	0.27
Q28	0.85	0.16	0.96	0.16	0.81	0.13	0.30
Q29	0.82	0.14	0.95	0.13	0.78	0.11	0.30
Q30	0.82	0.14	0.96	0.14	0.79	0.11	0.30
Q31	0.82	0.14	0.96	0.13	0.79	0.11	0.29
Q32	0.81	0.15	0.95	0.14	0.77	0.11	0.30
Q33	0.82	0.14	0.96	0.14	0.78	0.11	0.30
Q34	0.81	0.15	0.96	0.13	0.78	0.11	0.30
Q35	0.84	0.14	0.96	0.15	0.81	0.11	0.27
Q36	0.84	0.15	0.94	0.16	0.79	0.12	0.33

Users were invited to complete the questionnaire via email, which directed them to a 9-page web-based survey. They were able to review and change their responses. Each submission was linked to the same username used to access the CDSS. Participation was voluntary, with no incentives provided. Three reminder emails were sent over a 2-month period after software usage.

**Table 2.** Items used within the questionnaire to measure agreement to each statement on a Likert scale are categorized with effort expectancy (EE), perceived usefulness (PU), trust (T), information quality (IQ), evidence-based practices attitude (EB-A), clinical decision support system attitude (CDSS-A), system quality (SQ), facilitating conditions (FC), and intended use in the future (ITU).

Question	Category	Survey item
Q1	EE^a^	“It was easy to integrate the system into my workflow.”
Q2	EE	“I used the system while interacting with my patients.”
Q6	PU^b^	“The information was effective in helping me making decisions.”
Q7	PU	“The systems’ output is a useful source of information in addition to the NHG^c^ guidelines.”
Q8	PU	“Using the system improved the quality of patient care.”
Q9	PU	“Using the system improved patient outcomes.”
Q10	PU	“I believe that the tool was particularly useful for certain patient groups.”
Q13	PU	“I believe that using the tool, empowered my decision-making.”
Q14	T^d^	“I trusted the outputs provided by the system.”
Q15	IQ^e^/T	“The information accompanying the system’s output enabled me to understand how the predictions were made by the algorithm.”
Q16	IQ/T	“I used the knowledge acquired from the system in situations outside of the software.”
Q18	EB-A^f^	“I consider myself as a person that embraces change in health care.”
Q19	EB-A	“I would like to increase the use of evidence in my daily practice.”
Q20	EB-A	“I believe that analyzing observational data is a strong method to learn about patients underrepresented in classical medical research.”
Q21	EB-A	“I believe that the NHG guidelines are based on sufficient evidence.”
Q22	CDSS-A^g^	“I believe that working according to the NHG guidelines will lead to improved patient care.”
Q23	CDSS-A	“Overall, I believe that the advantages of clinical decision support systems outweigh the disadvantages.”
Q28	SQ^h^/FC^i^	“The system was sufficiently fast.”
Q29	SQ/FC	“I found the system interface too complex.”
Q30	SQ/FC	“The content of the software was readable and clearly organized.”
Q31	SQ/FC	“It was easy to enter the patient characteristics.”
Q32	SQ/FC	“It was easy to quickly extract the relevant information to support my decision-making.”
Q33	SQ/FC	“It was easy to access the analysis of previously entered patient data.”
Q34	FC	“Technical support was available when I needed it.”
Q36	ITU^j^	“Given that the most common diagnoses are incorporated in the decision support, I intend to use the system in the future.”
Q37	Satisfaction	“Overall, I am satisfied with the system.”

a EE: effort expectancy.

b PU: perceived usefulness.

c NHG: Dutch Association of General Practitioners guideline.

d T: trust.

e IQ: information quality.

f EB-A: evidence-based practices attitude.

g CDSS-A: clinical decision support system attitude.

h SQ: system quality.

i FC: facilitating condition.

j ITU: intended use in the future.

### Statistical Analysis

#### Usage Data

The present usage analysis extracted the extent of usage based on the number of entered patients, the total number of interactions with the software (General use), and the number of completed analyses with the software. The total number of interactions included the user’s performed actions, such as accessing patient data or logging in. The number of completed analyses refers to the number of patient files that were completed and filled out with the final response regarding the chosen medication. Descriptive statistics were used to estimate the overall usage over the trial period on the individual and practice levels.

#### Questionnaire

Quantitative questionnaire results are reported, and summary descriptives are provided. The free-text comments were independently reviewed, and themes were extracted by 2 researchers (WEH and JK) to limit any potential sources of error. Given the descriptive and exploratory nature of this usability study, analyses are limited to descriptive statistics; no hypothesis testing or inferential statistics were performed.

The datasets generated or analyzed during this study are available from the corresponding author on reasonable request.

### Ethical Considerations

The study protocol was reviewed and determined to meet the requirements for exemption from Ethics Committee (METC) review under the Dutch Medical Research Involving Human Subjects Act and to be in accordance with the Dutch Medical Treatment Act and the Dutch Data Protection Act. Patient privacy was protected throughout the study by design. All data were handled in accordance with applicable data protection regulations. No directly identifying information was accessed or included in the dataset used for this study, and no identifiable information is reported in this manuscript. Patients were informed about the use of their data for research purposes via the participating practices and were given the opportunity to opt out anonymously.

## Results

### Usage Data

Of the original 36 practices, 34 started with the implementation study (95%). Thirty-one practices continued using the tool throughout the pilot period, with a 9% dropout in the first 8 weeks.

Practices used the tool for an average of 40.14 consultations during the intervention period (SD 33.40; Table 3). Most of the activity was recorded within the first few weeks and slightly declined over time. A total of 153 (58%) users used the tool at least once, 98 (64%) of whom were assistants. On average, each user had 46 (SD 52.45) total system interactions and completed 8.42 (SD 10.64) analyses.

**Table 3.** Total number of clinical decision support system (CDSS) activities.

Usage type	In total	Per practice (n=36), mean (SD)	Range (minimum-maximum)
Patients with UTI^a^ entered	1365	40.14 (33.40)	1-123
Completed analyses	1150	33.82 (29.41)	1-108
General use	4085	120.15 (102.54)	3-414

a UTI: urinary tract infection.

### Questionnaire Statistics

Thirty out of 34 participating practices submitted at least one response, with practices submitting, on average, 2.23 responses (SD 1.43). In total, 67 health care professionals responded to the questionnaire, including 31 assistants and 36 GPs (Table 4) [32]. All pages of the questionnaire were completed by everyone.

**Table 4.** Characteristics of general practitioners (GPs) and assistants’ questionnaire respondents relative to registered Dutch GPs.

Characteristic	GPs, n (%)	Assistants, n (%)	Registered Dutch GPs, n
Sex			
Female	10 (27.78)	26 (83.87)	51
Male	24 (66.67)	1 (3.23)	49
NA^a^	2 (5.56)	4 (12.90)	0
Age			
<41 years	6 (16.67)	14 (45.16)	25
>41 years	28 (77.78)	14 (45.16)	75
NA^a^	2 (5.56)	3 (9.68)	0
Years of experience			
<1	1 (2.78)	0 (0.00)	—^b^
1‐5	2 (5.56)	5 (16.13)	—
6‐10	3 (8.33)	9 (29.03)	—
11‐20	16 (44.44)	6 (19.35)	—
21‐30	9 (25.00)	7 (22.58)	—
>30	4 (11.11)	1 (3.23)	—
NA^a^	1 (2.78)	3 (9.68)	—
Practice size^c^			
One GP	2 (5.56)	5 (16.13)	35
2 GPs	6 (16.67)	7 (22.59)	43
>2 GPs	28 (77.78)	19 (61.29)	22
CDSS^d^ use			
Yes	16 (44.44)	1 (3.23)	—
No	19 (52.78)	26 (83.87)	—
NA^a^	1 (2.78)	4 (11.11)	—

a Not applicable/Preferred not to say.

b Not available

c Practice size is an estimate based on the number of operating GPs within a practice.

d CDSS: clinical decision support system.

### Evaluation Outcomes

#### Clinical Process and Usefulness

**Figure 2.**
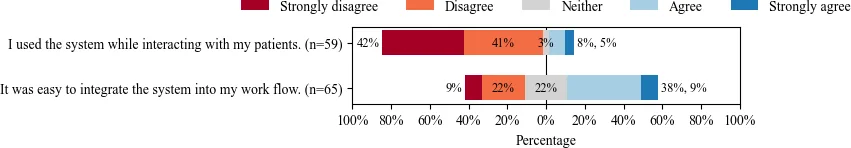
Respondents’ opinion on the effort expectancy of the software.

Thirty-one out of the total 65 (48%) respondents indicated that it was easy to integrate the tool into their clinical workflow, and 8 out of 59 (14%) respondents indicated that the tool was used during patient interaction (Figure 2).

**Figure 3.**
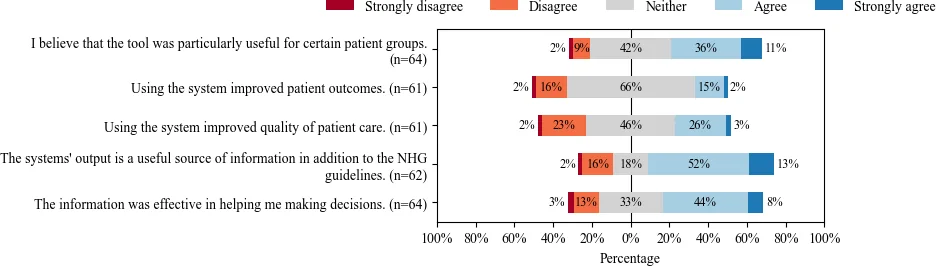
Respondents’ opinion on the usefulness and relevance of the software in clinical practice.

Overall, 40 out of 62 (65%) respondents evaluated the tool as useful in addition to the NHG guideline, and 33 out of 64 (52%) respondents agreed to the tool supporting them in their decision-making process. While the software’s usefulness is appreciated, the majority of respondents were not sure whether the tool improved patient care; 28 out of 61 (46%) respondents. Furthermore, the majority of respondents were not sure whether the tool improved patient outcomes; 40 out of 61 (66%) respondents (Figure 3).

#### Trust and Information Quality

**Figure 4.**
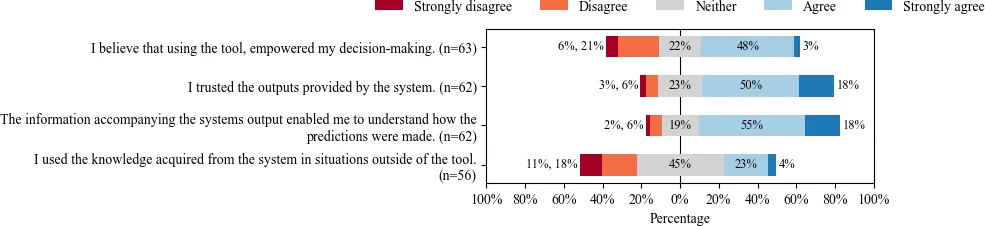
Respondents’ opinion on the user’s trust and the information quality of the software.

Sixty-eight percent of respondents indicated that they trusted the system’s output (Figure 4), namely 42 out of 62 respondents, which depicted the expected outcomes for each medication given specific patient characteristics. Additionally, 45 out of 62 (73%) respondents agreed that the output made it understandable how the algorithm came to its results.

#### General Attitudes

**Figure 5.**
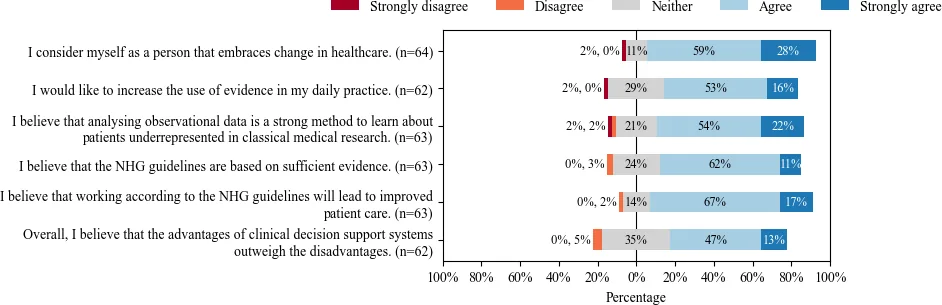
Respondents’ opinions on their own personal attitudes.

Forty-three out of 62 (69%) respondents indicated that they would like to increase the use of evidence in their daily practice, with 48 out of 63 (76%) respondents indicating that the use of observational data can be a strong method to learn about patients underrepresented in medical research (Figure 5). Fifty-three out of 63 (84%) respondents agreed to the statement that working according to the official professional guidelines leads to improved patient care. In addition, 46 out of 63 (73%) respondents believe that these guidelines are based on sufficient evidence. Fifty-six out of 64 (88%) respondents indicated that they embrace changes in health care. Finally, 37 out of 62 (60%) respondents agreed with the statement that clinical decision support tools have more advantages than disadvantages, while 22 out of these 62 (35%) respondents neither agreed nor disagreed with this statement.

#### System Evaluation

**Figure 6.**
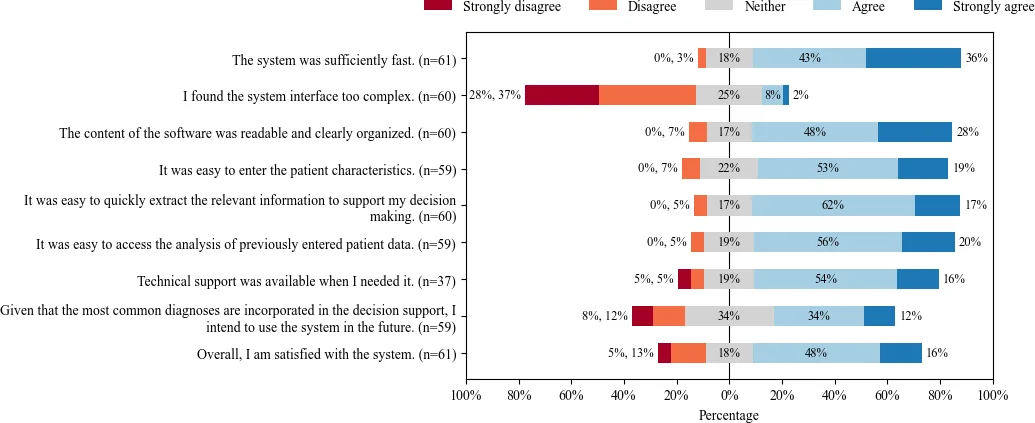
Respondents’ opinions on the system’s quality, their intention to use it, and their satisfaction with the software.

The overall system evaluation was positive, with 48 out of 61 (79%) respondents agreeing to the system being sufficiently fast and 46 out of 60 (77%) respondents agreeing to the system being clearly organized with an easy-to-understand interface (Figure 6). Furthermore, 47 out of 60 (78%) respondents indicated that it was easy to extract the relevant information. Forty-two out of 59 (71%) respondents agreed that it was easy to enter the patient characteristics. Thirty-nine of 61 (64%) respondents indicated to be satisfied with the decision support software, and 27 of 59 (46%) respondents stated that they would use the software in the future if major diseases were incorporated (Figure 6).

#### Free Text Comments

There were 4 free-text fields in the questionnaire that allowed users to further elaborate on some of their responses. The first question requested users to further elaborate on how the software was integrated into their daily activities. The main theme that emerged was that the use of the software required too much additional work and was too time-consuming. A second theme that emerged was regarding how the tool was used. It was stated that it was mostly assistants who interacted with the software by entering the patients. The presented information was then used by the GP. This also resulted in redistribution of work and extra discussion between doctor and assistant.

The second question, concerning the users’ main intentions to use the system, was answered by 47 out of 67 respondents (70%). The most frequently stated theme was the opportunity of added informative value that the users saw in decision support systems. The second theme that emerged was the potential of improved care through the delivery of more personalized treatments.

The final question requested further suggestions to improve the software, with the most common suggestions related to a request to integrate the tool into the EHR systems. Finally, respondents suggested the added value of additional training on how the software should be used well.

## Discussion

### Principal Results

The main results were that the CDSS was used frequently throughout the implementation period and that users perceived the information as effective in supporting decision-making and as a useful addition to existing professional guidelines. Participants also reported trusting the information, and the additional contextual information about the patient groups underlying predictions helped them understand how expected treatment outcomes were generated. This transparency may facilitate effective collaboration between clinicians and decision support. Participants stressed that the added informative value and potential improvement of patient outcomes are reasons to use a similar CDSS, which is in line with the relatively low dropout rate of 9%. Participants were, however, undecided about whether the CDSS indeed improved quality of care and patient outcomes during the implementation study. This could indicate either a discrepancy in the results of our study or imply the complexity of assessing the impact of a decision support tool on the quality of care for a physician. The fact that the CDSS was associated with a statistically significant improvement in patient outcomes during the intervention period suggests that the uncertainty may stem from the inherent complexity of evaluating the impact of such tools on quality of care [9].

Furthermore, participants clearly stressed the tool was easy to use and to retrieve and understand the information from the tool. Nevertheless, participants indicate that the use of the software required too much additional work and was too time-consuming. It was repeatedly mentioned that IT integration would facilitate a better match with this workflow. The fact that the use of the tool felt like a lot of extra work in practice seems to be the main reason for lack of use and adoption. This may partly explain why some participants were not inclined to use this type of CDSS in the future. The perceived time investment has been shown to influence the adoption of CDSS in previous research [15,20]. Even minimal additional steps can be perceived as too much if the tool does not align closely with existing routines. This highlights the importance of designing CDSS tools that align closely with existing clinical workflows, thereby minimizing perceived time investment and supporting sustained adoption.

### Limitations

In general, participants stated they have a positive attitude toward change in health care. Most of the participants also stated that they would like to increase the amount of evidence in their daily practice and that they see an opportunity in the use of observational data for doing so. In addition, the participants indicated that the NHG guideline leads to better patient care, and the participants mostly stated that the NHG guideline is based on sufficient evidence. This gives us reason to conclude that the participant sample was relatively positive toward change and several ways of increasing evidence in their daily practice. This could indicate a selection bias in our sample. The fact that part of the practice recruitment took place via care groups and thus not through the initiative of some of the practices themselves, this bias is possibly mitigated partly. Furthermore, although the response rate at practice level was very high (30 out of 34 practices), the response rate of the survey was substantially lower as compared to the total number of participants of the implementation study (67 out of 265 users). This means that the results of the survey could endure a response bias as well.

The use of the survey enables us to obtain relatively unbiased results from a large number of users. In the construction of the questionnaire, the UTAUT model was used, allowing the structure of the results and additional topics of specific interest were added. However, our particular survey was not fully validated before conduction. Doing an extensive pilot with a group of GPs that were not part of the study but were familiar with the software helped in assuring that the questions were interpreted and understood well. To further guarantee the reliability, validity, and quality of the survey items, SQP was used.

Another point of attention of the research is that the assistants had not been involved in the design phase of the CDSS, whereas they played an important role in the treatment of the UTIs in practice. In many participating practices, much of the work was done by assistants. As such, their role in the care path should not have been underestimated and future design should ensure that all potential user groups are included from the beginning. The different uses of the tool for assistants and GPs led to a redistribution of work, which resulted in less work. Nevertheless, evaluating and comparing the results between GP and assistant did not yield significant differences.

### Conclusions

In general, the users were very open to research and wanted to be involved in the development and evaluation of intelligent clinical decision support. Thorough research on the desires and concerns of the end user is needed to develop clinically relevant, usable, and safe decision support systems. An important aspect of such research is the impact on the trust of the information by the user. Trust in the CDSS is a key factor in ensuring effective collaboration between physicians and ML and ultimately in achieving optimal clinical outcomes. However, trust should not be maximized uncritically, as excessive trust can also become a significant pitfall [33]. It is important that, as for all medical knowledge a doctor has access to, the information is approached critically and is continuously being evaluated and monitored on quality. Large trust in the system could be at the cost of this critical view, and a noteworthy risk is that doctors are less well equipped to evaluate this new source of information as opposed to other sources of clinical information and as such might fall for the “automation bias” [24]. Automation bias happens when users become less vigilant in processing the information and trust it uncritically. For example, it has been shown that with the introduction of a computer algorithm that supported computer-aided detection in mammography, the performance of radiologists decreased, as the algorithm provided incorrect classifications [24,25]. During this study, for example, the users have indicated that they trust the system well but did not ask questions about the underlying mathematics or technology of the system, while we would have liked to see that users would have required more information before accepting and trusting the recommendation. However, the trust in the system could have also been a result of the conscious design choice to use relatively simple ML methods, allowing for extensive explainability, such as showing the explicit group of patients in the training dataset on which the predictions were based [9]. Such transparency has been shown to strongly support a positive relationship between ML and physicians [33].

Next to trust in the methodology, there were also almost no questions asked about potential conflicts of interests by the manufacturer of the CDSS. It might well be that manufacturers have specific interests in selling algorithms that enhance other products that they sell, like algorithms that support in- and detubation timing, that enhance the value of respirators. Or manufacturers that have clear interests in reducing costs more than in improving the quality of care. For safe adoption on a large scale, these critical and sometimes ethical questions are important not to neglect.

## Supplementary material

10.2196/80527 Checklist 1 CONSORT-EHEALTH (V 1.6.1) checklist.

## Abbreviations

CDSS: Clinical Decision Support System
DCGP: Dutch College of General Practitioners
EHR: electronic health record
GP: general practitioner
LUMC: Leiden University Medical Center
ML: machine learning
NHG: Dutch Association of General Practitioners
NIVEL: Netherlands Institute for Health Services Research
RCT: randomized controlled trial
SQP: survey quality predictor
UTAUT: Unified Theory of Acceptance and Use of Technology
UTI: urinary tract infection
