# Supplementary material for: Usability and Usefulness of Machine Learning–Based Clinical Decision Support Software in Primary Care: Survey of Users in a Prospective Observational Study
Source: JMIR Med Inform. 2026 May 27;14:e80527. doi: 10.2196/80527 (PMC13564043; doi:10.2196/80527)
Supplement: Supplementary file 1 [file medinform-v14-e80527-s001.pdf]

CONSORT-EHEALTH (V 1.6.1) - Submission/Publication Form

The CONSORT-EHEALTH checklist is intended for authors of randomized trials evaluating web-based and Internet-based applications/interventions, including mobile interventions, electronic games (incl multiplayer games), social media, certain telehealth applications, and other interactive and/or networked electronic applications. Some of the items (e.g. all subitems under item 5 - description of the intervention) may also be applicable for other study designs.

The goal of the CONSORT EHEALTH checklist and guideline is to be
a) a guide for reporting for authors of RCTs,
b) to form a basis for appraisal of an ehealth trial (in terms of validity)

CONSORT-EHEALTH items/subitems are MANDATORY reporting items for studies published in the Journal of Medical Internet Research and other journals / scientific societies endorsing the checklist.

Items numbered 1., 2., 3., 4a., 4b etc are original CONSORT or CONSORT-NPT (non-pharmacologic treatment) items.
Items with Roman numerals (i., ii, iii, iv etc.) are CONSORT-EHEALTH extensions/clarifications.

As the CONSORT-EHEALTH checklist is still considered in a formative stage, we would ask that you also RATE ON A SCALE OF 1-5 how important/useful you feel each item is FOR THE PURPOSE OF THE CHECKLIST and reporting guideline (optional).

Mandatory reporting items are marked with a red *.
In the textboxes, either copy & paste the relevant sections from your manuscript into this form - please include any quotes from your manuscript in QUOTATION MARKS,
or answer directly by providing additional information not in the manuscript, or elaborating on why the item was not relevant for this study.

YOUR ANSWERS WILL BE PUBLISHED AS A SUPPLEMENTARY FILE TO YOUR PUBLICATION IN JMIR AND ARE CONSIDERED PART OF YOUR PUBLICATION (IF ACCEPTED).
Please fill in these questions diligently. Information will not be copyedited, so please use proper spelling and grammar, use correct capitalization, and avoid abbreviations.

DO NOT FORGET TO SAVE AS PDF _AND_ CLICK THE SUBMIT BUTTON SO YOUR ANSWERS ARE IN OUR DATABASE !!!

Citation Suggestion (if you append the pdf as Appendix we suggest to cite this paper in the caption):
Eysenbach G, CONSORT-EHEALTH Group
CONSORT-EHEALTH: Improving and Standardizing Evaluation Reports of Web-based and Mobile Health Interventions
J Med Internet Res 2011;13(4):e126
URL: http://www.jmir.org/2011/4/e126/
doi: 10.2196/jmir.1923
PMID: 22209829

wherter@gmail.com Switch account

Not shared

Draft saved

* Indicates required question

Your name *

First Last

Willem Herter

Primary Affiliation (short), City, Country *

University of Toronto, Toronto, Canada

Leiden University Medical Center

Your e-mail address *

abc@gmail.com

w.e.herter@lumc.nl

Title of your manuscript *

Provide the (draft) title of your manuscript.

Usability and usefulness of machine learning based clinical decision support software in primary care: Survey of users in prospective observational study

Name of your App/Software/Intervention *

If there is a short and a long/alternate name, write the short name first and add the long name in brackets.

Pacmed Clinical Decision Support System

Evaluated Version (if any)

e.g. "V1", "Release 2017-03-01", "Version 2.0.27913"

Your answer

Language(s) *

What language is the intervention/app in? If multiple languages are available, separate by comma (e.g. "English, French")

Dutch

URL of your Intervention Website or App

e.g. a direct link to the mobile app on app in appstore (itunes, Google Play), or URL of the website. If the intervention is a DVD or hardware, you can also link to an Amazon page.

Your answer

URL of an image/screenshot (optional)

Your answer

Accessibility *

Can an enduser access the intervention presently?

- ○ access is free and open
- ○ access only for special usergroups, not open
- ○ access is open to everyone, but requires payment/subscription/in-app purchases
- ◉ app/intervention no longer accessible
- ○ Other:

Primary Medical Indication/Disease/Condition *

e.g. "Stress", "Diabetes", or define the target group in brackets after the condition, e.g. "Autism (Parents of children with)", "Alzheimers (Informal Caregivers of)"

Urinary Tract Infections

Primary Outcomes measured in trial *

comma-separated list of primary outcomes reported in the trial

The primary outcome of interest was the differ

Secondary/other outcomes

Are there any other outcomes the intervention is expected to affect?

A survey was constructed using the unified theory of technology acceptance model (UTAUT) and sent to 265 general practitioners and assistants shortly after the implementation period. Furthermore, to investigate usage behaviour with Pacmed's clinical decision support software data of the user's interaction with the software was analysed.

Recommended "Dose" *

What do the instructions for users say on how often the app should be used?

- (selected) Approximately Daily
- Approximately Weekly
- Approximately Monthly
- Approximately Yearly
- "as needed"
- Other:

Approx. Percentage of Users (starters) still using the app as recommended after 3 months *

- [x] unknown / not evaluated
- [ ] 0-10%
- [ ] 11-20%
- [ ] 21-30%
- [ ] 31-40%
- [ ] 41-50%
- [ ] 51-60%
- [ ] 61-70%
- [ ] 71%-80%
- [ ] 81-90%
- [ ] 91-100%
- [ ] Other:

Overall, was the app/intervention effective? *

- [x] yes: all primary outcomes were significantly better in intervention group vs control
- [ ] partly: SOME primary outcomes were significantly better in intervention group vs control
- [ ] no statistically significant difference between control and intervention
- [ ] potentially harmful: control was significantly better than intervention in one or more outcomes
- [ ] inconclusive: more research is needed
- [ ] Other:

Article Preparation Status/Stage *

At which stage in your article preparation are you currently (at the time you fill in this form)

- ( ) not submitted yet - in early draft status
- ( ) not submitted yet - in late draft status, just before submission
- ( ) submitted to a journal but not reviewed yet
- ( ) submitted to a journal and after receiving initial reviewer comments
- ( ) submitted to a journal and accepted, but not published yet
- ( ) published
- (x) Other: Primary outcome article was published by JMIR, this paper is on som

Journal *

If you already know where you will submit this paper (or if it is already submitted), please provide the journal name (if it is not JMIR, provide the journal name under "other")

- ( ) not submitted yet / unclear where I will submit this
- (x) Journal of Medical Internet Research (JMIR)
- ( ) JMIR mHealth and UHealth
- ( ) JMIR Serious Games
- ( ) JMIR Mental Health
- ( ) JMIR Public Health
- ( ) JMIR Formative Research
- ( ) Other JMIR sister journal
- ( ) Other:

Is this a full powered effectiveness trial or a pilot/feasibility trial? *

- (x) Pilot/feasibility
- ( ) Fully powered

Manuscript tracking number *

If this is a JMIR submission, please provide the manuscript tracking number under "other" (The ms tracking number can be found in the submission acknowledgement email, or when you login as author in JMIR. If the paper is already published in JMIR, then the ms tracking number is the four-digit number at the end of the DOI, to be found at the bottom of each published article in JMIR)

- (selected) no ms number (yet) / not (yet) submitted to / published in JMIR
- Other:

TITLE AND ABSTRACT

1a) TITLE: Identification as a randomized trial in the title

1a) Does your paper address CONSORT item 1a? *

I.e does the title contain the phrase "Randomized Controlled Trial"? (if not, explain the reason under "other")

- yes
- (selected) Other: Prospective observational study, since it cannot qualify as an fully ran

1a-i) Identify the mode of delivery in the title

Identify the mode of delivery. Preferably use "web-based" and/or "mobile" and/or "electronic game" in the title. Avoid ambiguous terms like "online", "virtual", "interactive". Use "Internet-based" only if Intervention includes non-web-based Internet components (e.g. email), use "computer-based" or "electronic" only if offline products are used. Use "virtual" only in the context of "virtual reality" (3-D worlds). Use "online" only in the context of "online support groups". Complement or substitute product names with broader terms for the class of products (such as "mobile" or "smart phone" instead of "iphone"), especially if the application runs on different platforms.

	1	2	3	4	5	
subitem not at all important	○	●	○	○	○	essential

Clear selection

Does your paper address subitem 1a-i? *

Copy and paste relevant sections from manuscript title (include quotes in quotation marks "like this" to indicate direct quotes from your manuscript), or elaborate on this item by providing additional information not in the ms, or briefly explain why the item is not applicable/relevant for your study

Participants were recruited on a care group level and such not individually randomised.

1a-ii) Non-web-based components or important co-interventions in title

Mention non-web-based components or important co-interventions in title, if any (e.g., “with telephone support”).

	1	2	3	4	5	
subitem not at all important	◉	○	○	○	○	essential

Clear selection

Does your paper address subitem 1a-ii?

Copy and paste relevant sections from manuscript title (include quotes in quotation marks "like this" to indicate direct quotes from your manuscript), or elaborate on this item by providing additional information not in the ms, or briefly explain why the item is not applicable/relevant for your study

Your answer

1a-iii) Primary condition or target group in the title

Mention primary condition or target group in the title, if any (e.g., “for children with Type I Diabetes”) Example: A Web-based and Mobile Intervention with Telephone Support for Children with Type I Diabetes: Randomized Controlled Trial

	1	2	3	4	5	
subitem not at all important	○	○	◉	○	○	essential

Clear selection

Does your paper address subitem 1a-iii? *

Copy and paste relevant sections from manuscript title (include quotes in quotation marks "like this" to indicate direct quotes from your manuscript), or elaborate on this item by providing additional information not in the ms, or briefly explain why the item is not applicable/relevant for your study

We have now excluded this since the study is a more general one; disease agnostic factors impacting usability. And the title would become too long.

1b) ABSTRACT: Structured summary of trial design, methods, results, and conclusions

NPT extension: Description of experimental treatment, comparator, care providers, centers, and blinding status.

1b-i) Key features/functionalities/components of the intervention and comparator in the METHODS section of the ABSTRACT

Mention key features/functionalities/components of the intervention and comparator in the abstract. If possible, also mention theories and principles used for designing the site. Keep in mind the needs of systematic reviewers and indexers by including important synonyms. (Note: Only report in the abstract what the main paper is reporting. If this information is missing from the main body of text, consider adding it)

	1	2	3	4	5	
subitem not at all important	(x)	( )	( )	( )	( )	essential

Clear selection

Does your paper address subitem 1b-i? *

Copy and paste relevant sections from the manuscript abstract (include quotes in quotation marks "like this" to indicate direct quotes from your manuscript), or elaborate on this item by providing additional information not in the ms, or briefly explain why the item is not applicable/relevant for your study

"In collaboration with NIVEL and LUMC, Pacmed developed the CDSS. The cohort was mostly recruited on caregroup level, and all the practices within a caregroup were obliged to participate. Health insurers funded the research partly. The practices participated in the implementation study for a period of four months. A survey was constructed using the unified theory of technology acceptance model (UTAUT) and sent to 265 general practitioners and assistants shortly after the implementation period. Furthermore, usage data was analysed."

1b-ii) Level of human involvement in the METHODS section of the ABSTRACT

Clarify the level of human involvement in the abstract, e.g., use phrases like “fully automated” vs. “therapist/nurse/care provider/physician-assisted” (mention number and expertise of providers involved, if any). (Note: Only report in the abstract what the main paper is reporting. If this information is missing from the main body of text, consider adding it)

	1	2	3	4	5	
subitem not at all important	○	○	●	○	○	essential

Clear selection

Does your paper address subitem 1b-ii?

Copy and paste relevant sections from the manuscript abstract (include quotes in quotation marks "like this" to indicate direct quotes from your manuscript), or elaborate on this item by providing additional information not in the ms, or briefly explain why the item is not applicable/relevant for your study

This is a good point, and the fact that the intervention is called a Decision Support System might qualify for 1b-ii?

1b-iii) Open vs. closed, web-based (self-assessment) vs. face-to-face assessments in the METHODS section of the ABSTRACT

Mention how participants were recruited (online vs. offline), e.g., from an open access website or from a clinic or a closed online user group (closed usergroup trial), and clarify if this was a purely web-based trial, or there were face-to-face components (as part of the intervention or for assessment). Clearly say if outcomes were self-assessed through questionnaires (as common in web-based trials). Note: In traditional offline trials, an open trial (open-label trial) is a type of clinical trial in which both the researchers and participants know which treatment is being administered. To avoid confusion, use “blinded” or “unblinded” to indicated the level of blinding instead of “open”, as “open” in web-based trials usually refers to “open access” (i.e. participants can self-enrol). (Note: Only report in the abstract what the main paper is reporting. If this information is missing from the main body of text, consider adding it)

	1	2	3	4	5	
subitem not at all important	●	○	○	○	○	essential

Clear selection

Does your paper address subitem 1b-iii?

Copy and paste relevant sections from the manuscript abstract (include quotes in quotation marks "like this" to indicate direct quotes from your manuscript), or elaborate on this item by providing additional information not in the ms, or briefly explain why the item is not applicable/relevant for your study

This has been discussed more elaborately in Part 1, the article on clinical impact (doi: 10.2196/27795) already published by JMIR, where we have specified how the users were recruited for the prospective study. Now, in the Methods section we solely discuss how we have recruited survey participants: "A survey was constructed using the unified theory of technology acceptance model (UTAUT) and sent to 265 general practitioners and assistants shortly after the implementation period."

1b-iv) RESULTS section in abstract must contain use data

Report number of participants enrolled/assessed in each group, the use/uptake of the intervention (e.g., attrition/adherence metrics, use over time, number of logins etc.), in addition to primary/secondary outcomes. (Note: Only report in the abstract what the main paper is reporting. If this information is missing from the main body of text, consider adding it)

	1	2	3	4	5	
subitem not at all important	○	○	◉	○	○	essential

Clear selection

Does your paper address subitem 1b-iv?

Copy and paste relevant sections from the manuscript abstract (include quotes in quotation marks "like this" to indicate direct quotes from your manuscript), or elaborate on this item by providing additional information not in the ms, or briefly explain why the item is not applicable/relevant for your study

"Thirty out of 34 participating practices (88%) submitted at least one response with practices submitting on average 2.23 responses (SD = 1.43). The CDSS was used continuously by all practices. 31 practices continued using the tool throughout the pilot period, with a 9% dropout in the first 8 weeks."

1b-v) CONCLUSIONS/DISCUSSION in abstract for negative trials

Conclusions/Discussions in abstract for negative trials: Discuss the primary outcome - if the trial is negative (primary outcome not changed), and the intervention was not used, discuss whether negative results are attributable to lack of uptake and discuss reasons. (Note: Only report in the abstract what the main paper is reporting. If this information is missing from the main body of text, consider adding it)

	1	2	3	4	5	
subitem not at all important	○	○	◉	○	○	essential

Clear selection

Does your paper address subitem 1b-v?

Copy and paste relevant sections from the manuscript abstract (include quotes in quotation marks "like this" to indicate direct quotes from your manuscript), or elaborate on this item by providing additional information not in the ms, or briefly explain why the item is not applicable/relevant for your study

"The CDSS was perceived as trustworthy and easy to use. However, users were unable to determine whether the CDSS improved patient outcome. Also, the study could have benefitted from including assistants as well as general practitioners more in the design phase of the software. Assistants play an important role in the treatment of urinary tract infections, and to design the software that fits the current process of care well, seems to have potential of positive effects on the perceived time investment of the use of the tool. Lastly, all the users seem to be very highly motivated to contribute to further research in this field and indicate to be willing to embrace change in the way healthcare is provided. This could, however, also signal selection bias in our study."

INTRODUCTION

2a) In INTRODUCTION: Scientific background and explanation of rationale

2a-i) Problem and the type of system/solution

Describe the problem and the type of system/solution that is object of the study: intended as stand-alone intervention vs. incorporated in broader health care program? Intended for a particular patient population? Goals of the intervention, e.g., being more cost-effective to other interventions, replace or complement other solutions? (Note: Details about the intervention are provided in “Methods” under 5)

	1	2	3	4	5	
subitem not at all important	○	○	○	●	○	essential

Clear selection

Does your paper address subitem 2a-i? *

Copy and paste relevant sections from the manuscript (include quotes in quotation marks "like this" to indicate direct quotes from your manuscript), or elaborate on this item by providing additional information not in the ms, or briefly explain why the item is not applicable/relevant for your study

"In a collaboration of NIVEL, LUMC and Pacmed, a data driven CDSS to aid the efficient treatment of UTIs was built. Aim was to support GP’s and patients with UTI treatment decisions. This CDSS was implemented in 36 general practices and was associated with a statistically significant improvement in treatment success [8]."

2a-ii) Scientific background, rationale: What is known about the (type of) system

Scientific background, rationale: What is known about the (type of) system that is the object of the study (be sure to discuss the use of similar systems for other conditions/diagnoses, if appropiate), motivation for the study, i.e. what are the reasons for and what is the context for this specific study, from which stakeholder viewpoint is the study performed, potential impact of findings [2]. Briefly justify the choice of the comparator.

	1	2	3	4	5	
subitem not at all important	○	○	○	●	○	essential

Clear selection

Does your paper address subitem 2a-ii? *

Copy and paste relevant sections from the manuscript (include quotes in quotation marks "like this" to indicate direct quotes from your manuscript), or elaborate on this item by providing additional information not in the ms, or briefly explain why the item is not applicable/relevant for your study

"Artificial intelligence (AI) applications are increasingly being developed for use in healthcare, and a growing body of this work is published in peer-reviewed journals. However, only a small fraction of these applications are ultimately implemented in clinical practice, and their efficacy and usability in real-world settings are rarely evaluated [1].

The implementation of AI and data analytics in healthcare faces many challenges. One key barrier is the willingness of healthcare professionals to adopt clinical decision support systems (CDSS) and to engage with insights derived from electronic health record (EHR) data. To explore these implementation challenges, we studied the introduction of a Clinical Decision Support System (CDSS) designed to support general practitioners (GPs) in selecting appropriate treatments for patients with urinary tract infections (UTIs)."

2b) In INTRODUCTION: Specific objectives or hypotheses

Does your paper address CONSORT subitem 2b? *

Copy and paste relevant sections from the manuscript (include quotes in quotation marks "like this" to indicate direct quotes from your manuscript), or elaborate on this item by providing additional information not in the ms, or briefly explain why the item is not applicable/relevant for your study

"Many reasons for the lack of CDSS adoption have been identified. More specifically, adoption of technologies within the healthcare domain have been driven by the user's perception of their usefulness, which included the relevance of information provided by the system, degree of applicability to patient characteristics, trust in the system, and its fit into the workflow of clinical practice [17–23]. Also, the user's attitudes towards changes in healthcare and evidence based practice are significant factors in adopting CDSS in practice [22,24]. These reasons only partly explain the limited adoption of CDSS. Therefore it is crucial to investigate and recognize the problems associated with technology adoption by healthcare professionals, to successfully implement decision support in clinical practice. Furthermore, more elaborate research on possible risks and pitfalls implementing decision support should be done."

METHODS

3a) Description of trial design (such as parallel, factorial) including allocation ratio

Does your paper address CONSORT subitem 3a? *

Copy and paste relevant sections from the manuscript (include quotes in quotation marks "like this" to indicate direct quotes from your manuscript), or elaborate on this item by providing additional information not in the ms, or briefly explain why the item is not applicable/relevant for your study

"The research is a quantitative interventional design. The cohort consists of 36 practices encompassing 264 users in total, 118 general practitioners and 147 assistants."

3b) Important changes to methods after trial commencement (such as eligibility criteria), with reasons

Does your paper address CONSORT subitem 3b? *

Copy and paste relevant sections from the manuscript (include quotes in quotation marks "like this" to indicate direct quotes from your manuscript), or elaborate on this item by providing additional information not in the ms, or briefly explain why the item is not applicable/relevant for your study

Subitem 3b is deemed non applicable in our case, since there were no changes after commencement.

3b-i) Bug fixes, Downtimes, Content Changes

Bug fixes, Downtimes, Content Changes: ehealth systems are often dynamic systems. A description of changes to methods therefore also includes important changes made on the intervention or comparator during the trial (e.g., major bug fixes or changes in the functionality or content) (5-iii) and other “unexpected events” that may have influenced study design such as staff changes, system failures/downtimes, etc. [2].

	1	2	3	4	5	
subitem not at all important	○	○	○	○	◉	essential

Clear selection

Does your paper address subitem 3b-i?

Copy and paste relevant sections from the manuscript (include quotes in quotation marks "like this" to indicate direct quotes from your manuscript), or elaborate on this item by providing additional information not in the ms, or briefly explain why the item is not applicable/relevant for your study

Subitem 3b-i is deemed non applicable in our case, since there were no changes from this kind in our study.

4a) Eligibility criteria for participants

Does your paper address CONSORT subitem 4a? *

Copy and paste relevant sections from the manuscript (include quotes in quotation marks "like this" to indicate direct quotes from your manuscript), or elaborate on this item by providing additional information not in the ms, or briefly explain why the item is not applicable/relevant for your study

"The cohort was mostly recruited on caregroup level, and all the practices within a caregroup were then obliged to participate."

4a-i) Computer / Internet literacy

Computer / Internet literacy is often an implicit "de facto" eligibility criterion - this should be explicitly clarified.

	1	2	3	4	5	
subitem not at all important	○	○	○	○	◉	essential

Clear selection

Does your paper address subitem 4a-i?

Copy and paste relevant sections from the manuscript (include quotes in quotation marks "like this" to indicate direct quotes from your manuscript), or elaborate on this item by providing additional information not in the ms, or briefly explain why the item is not applicable/relevant for your study

Now it does: "Computer or internet literacy was not used as an explicit eligibility criterion."

4a-ii) Open vs. closed, web-based vs. face-to-face assessments:

Open vs. closed, web-based vs. face-to-face assessments: Mention how participants were recruited (online vs. offline), e.g., from an open access website or from a clinic, and clarify if this was a purely web-based trial, or there were face-to-face components (as part of the intervention or for assessment), i.e., to what degree got the study team to know the participant. In online-only trials, clarify if participants were quasi-anonymous and whether having multiple identities was possible or whether technical or logistical measures (e.g., cookies, email confirmation, phone calls) were used to detect/prevent these.

	1	2	3	4	5	
subitem not at all important	○	○	○	◉	○	essential

Clear selection

Does your paper address subitem 4a-ii? *

Copy and paste relevant sections from the manuscript (include quotes in quotation marks "like this" to indicate direct quotes from your manuscript), or elaborate on this item by providing additional information not in the ms, or briefly explain why the item is not applicable/relevant for your study

"This research on usability and perceived usefulness is a cross-sectional study using an online survey that was sent out to all 265 users after completion of the intervention period."

4a-iii) Information giving during recruitment

Information given during recruitment. Specify how participants were briefed for recruitment and in the informed consent procedures (e.g., publish the informed consent documentation as appendix, see also item X26), as this information may have an effect on user self-selection, user expectation and may also bias results.

	1	2	3	4	5	
subitem not at all important	○	○	◉	○	○	essential

Clear selection

Does your paper address subitem 4a-iii?

Copy and paste relevant sections from the manuscript (include quotes in quotation marks "like this" to indicate direct quotes from your manuscript), or elaborate on this item by providing additional information not in the ms, or briefly explain why the item is not applicable/relevant for your study

Your answer

4b) Settings and locations where the data were collected

Does your paper address CONSORT subitem 4b? *

Copy and paste relevant sections from the manuscript (include quotes in quotation marks "like this" to indicate direct quotes from your manuscript), or elaborate on this item by providing additional information not in the ms, or briefly explain why the item is not applicable/relevant for your study

"This research on usability and perceived usefulness is a cross-sectional study using an online survey that was sent out to all 265 users after completion of the intervention period." and "Use of the CDSS was recorded in the CDSS tool itself. The clinical results of the usage as well as the behaviour change of the practices during the implementation study is evaluated in another paper [8]."

4b-i) Report if outcomes were (self-)assessed through online questionnaires

Clearly report if outcomes were (self-)assessed through online questionnaires (as common in web-based trials) or otherwise.

	1	2	3	4	5	
subitem not at all important	○	○	○	◉	○	essential

Clear selection

Does your paper address subitem 4b-i? *

Copy and paste relevant sections from the manuscript (include quotes in quotation marks "like this" to indicate direct quotes from your manuscript), or elaborate on this item by providing additional information not in the ms, or briefly explain why the item is not applicable/relevant for your study

"Quantitative questionnaire results are reported and summary descriptives are provided. The free text comments were independently reviewed, and themes were extracted by two researchers (Herter and Khuc) to limit any potential sources of error." and "The present usage analysis extracted the extent of usage based on the number of entered patients, the total number of interactions with the software and the number of completed analyses with the software. The total number of interactions included the user's performed actions such as accessing of patient data or logins. The number of completed analysis refers to the number of patient files that were completed and filled out with the final response regarding the chosen medication. Descriptive statistics were used to estimate the overall usage over the trial period on individual and practice level."

4b-ii) Report how institutional affiliations are displayed

Report how institutional affiliations are displayed to potential participants [on ehealth media], as affiliations with prestigious hospitals or universities may affect volunteer rates, use, and reactions with regards to an intervention.(Not a required item – describe only if this may bias results)

	1	2	3	4	5	
subitem not at all important	○	○	○	●	○	essential

Clear selection

Does your paper address subitem 4b-ii?

Copy and paste relevant sections from the manuscript (include quotes in quotation marks "like this" to indicate direct quotes from your manuscript), or elaborate on this item by providing additional information not in the ms, or briefly explain why the item is not applicable/relevant for your study

Your answer

5) The interventions for each group with sufficient details to allow replication, including how and when they were actually administered

5-i) Mention names, credential, affiliations of the developers, sponsors, and owners

Mention names, credential, affiliations of the developers, sponsors, and owners [6] (if authors/evaluators are owners or developer of the software, this needs to be declared in a "Conflict of interest" section or mentioned elsewhere in the manuscript).

	1	2	3	4	5	
subitem not at all important	○	○	○	◉	○	essential

Clear selection

Does your paper address subitem 5-i?

Copy and paste relevant sections from the manuscript (include quotes in quotation marks "like this" to indicate direct quotes from your manuscript), or elaborate on this item by providing additional information not in the ms, or briefly explain why the item is not applicable/relevant for your study

"Herter is a PhD candidate at Leiden University Medical Center (LUMC) as well as a former director at Pacmed. Khuc worked at Pacmed during their contribution to the research and paper. Health insurers funded the research partly."

5-ii) Describe the history/development process

Describe the history/development process of the application and previous formative evaluations (e.g., focus groups, usability testing), as these will have an impact on adoption/use rates and help with interpreting results.

	1	2	3	4	5	
subitem not at all important	○	○	○	◉	○	essential

Clear selection

Does your paper address subitem 5-ii?

Copy and paste relevant sections from the manuscript (include quotes in quotation marks "like this" to indicate direct quotes from your manuscript), or elaborate on this item by providing additional information not in the ms, or briefly explain why the item is not applicable/relevant for your study

"The decision support software was developed as a collaborative effort between NIVEL, LUMC and Pacmed. The software uses algorithms based on the analysis of data from NIVEL Primary Care Database, that included a total of 122.203 urinary tract infections [27]. The software interface was developed in close collaboration with several primary care physicians through user tests and expert groups."

5-iii) Revisions and updating

Revisions and updating. Clearly mention the date and/or version number of the application/intervention (and comparator, if applicable) evaluated, or describe whether the intervention underwent major changes during the evaluation process, or whether the development and/or content was “frozen” during the trial. Describe dynamic components such as news feeds or changing content which may have an impact on the replicability of the intervention (for unexpected events see item 3b).

	1	2	3	4	5	
subitem not at all important	○	○	○	○	○	essential

Does your paper address subitem 5-iii?

Copy and paste relevant sections from the manuscript (include quotes in quotation marks "like this" to indicate direct quotes from your manuscript), or elaborate on this item by providing additional information not in the ms, or briefly explain why the item is not applicable/relevant for your study

Your answer

5-iv) Quality assurance methods

Provide information on quality assurance methods to ensure accuracy and quality of information provided [1], if applicable.

	1	2	3	4	5	
subitem not at all important	○	○	○	◉	○	essential

Clear selection

Does your paper address subitem 5-iv?

Copy and paste relevant sections from the manuscript (include quotes in quotation marks "like this" to indicate direct quotes from your manuscript), or elaborate on this item by providing additional information not in the ms, or briefly explain why the item is not applicable/relevant for your study

This is mentioned in the other article, on clinical impact, which is already published (doi: 10.2196/27795).

5-v) Ensure replicability by publishing the source code, and/or providing screenshots/screen-capture video, and/or providing flowcharts of the algorithms used

Ensure replicability by publishing the source code, and/or providing screenshots/screen-capture video, and/or providing flowcharts of the algorithms used. Replicability (i.e., other researchers should in principle be able to replicate the study) is a hallmark of scientific reporting.

	1	2	3	4	5	
subitem not at all important	○	○	○	◉	○	essential

Clear selection

Does your paper address subitem 5-v?

Copy and paste relevant sections from the manuscript (include quotes in quotation marks "like this" to indicate direct quotes from your manuscript), or elaborate on this item by providing additional information not in the ms, or briefly explain why the item is not applicable/relevant for your study

"Figure 1. Decision support software. A- interface to enter patient characteristics B- presentation of expected outcomes and the recommendation of the NHG guidelines for a specific patient."

5-vi) Digital preservation

Digital preservation: Provide the URL of the application, but as the intervention is likely to change or disappear over the course of the years; also make sure the intervention is archived (Internet Archive, webcitation.org, and/or publishing the source code or screenshots/videos alongside the article). As pages behind login screens cannot be archived, consider creating demo pages which are accessible without login.

	1	2	3	4	5	
subitem not at all important	○	○	○	○	○	essential

Does your paper address subitem 5-vi?

Copy and paste relevant sections from the manuscript (include quotes in quotation marks "like this" to indicate direct quotes from your manuscript), or elaborate on this item by providing additional information not in the ms, or briefly explain why the item is not applicable/relevant for your study

Your answer

5-vii) Access

Access: Describe how participants accessed the application, in what setting/context, if they had to pay (or were paid) or not, whether they had to be a member of specific group. If known, describe how participants obtained "access to the platform and Internet" [1]. To ensure access for editors/reviewers/readers, consider to provide a "backdoor" login account or demo mode for reviewers/readers to explore the application (also important for archiving purposes, see vi).

	1	2	3	4	5	
subitem not at all important	○	○	○	◉	○	essential

Clear selection

Does your paper address subitem 5-vii? *

Copy and paste relevant sections from the manuscript (include quotes in quotation marks "like this" to indicate direct quotes from your manuscript), or elaborate on this item by providing additional information not in the ms, or briefly explain why the item is not applicable/relevant for your study

"Users were requested to enter patients' characteristics, and the CDSS then provided the users with a bar chart of the expected outcomes for the relevant treatment options based on similar patients in the database. Next to that, the relevant part of the NHG guideline was presented. Additional information supporting the expected outcomes could be retrieved by clicking on a treatment option. This information included the number of patients the analysis was based on, in addition to an age band of these patients and other clinically relevant features (Figure 1)."

5-viii) Mode of delivery, features/functionalities/components of the intervention and comparator, and the theoretical framework

Describe mode of delivery, features/functionalities/components of the intervention and comparator, and the theoretical framework [6] used to design them (instructional strategy [1], behaviour change techniques, persuasive features, etc., see e.g., [7, 8] for terminology). This includes an in-depth description of the content (including where it is coming from and who developed it) [1]," whether [and how] it is tailored to individual circumstances and allows users to track their progress and receive feedback" [6]. This also includes a description of communication delivery channels and – if computer-mediated communication is a component – whether communication was synchronous or asynchronous [6]. It also includes information on presentation strategies [1], including page design principles, average amount of text on pages, presence of hyperlinks to other resources, etc. [1].

	1	2	3	4	5	
subitem not at all important	○	○	○	◉	○	essential

Clear selection

Does your paper address subitem 5-viii? *

Copy and paste relevant sections from the manuscript (include quotes in quotation marks "like this" to indicate direct quotes from your manuscript), or elaborate on this item by providing additional information not in the ms, or briefly explain why the item is not applicable/relevant for your study

This item seems out of scope for this paper, which discusses the results of the survey questionnaire mostly.

5-ix) Describe use parameters

Describe use parameters (e.g., intended "doses" and optimal timing for use). Clarify what instructions or recommendations were given to the user, e.g., regarding timing, frequency, heaviness of use, if any, or was the intervention used ad libitum.

	1	2	3	4	5	
subitem not at all important	○	○	○	◉	○	essential

Clear selection

Does your paper address subitem 5-ix?

Copy and paste relevant sections from the manuscript (include quotes in quotation marks "like this" to indicate direct quotes from your manuscript), or elaborate on this item by providing additional information not in the ms, or briefly explain why the item is not applicable/relevant for your study

"Usage behaviour was continuously recorded as users interacted with the software, which includes the time of service, the number of logins, as well as the activities mentioned above (creating of new patient files, editing of patient files, accessing patient files, medications suggested before and after results presentation as well as provision of feedback to the developers)."

5-x) Clarify the level of human involvement

Clarify the level of human involvement (care providers or health professionals, also technical assistance) in the e-intervention or as co-intervention (detail number and expertise of professionals involved, if any, as well as “type of assistance offered, the timing and frequency of the support, how it is initiated, and the medium by which the assistance is delivered”. It may be necessary to distinguish between the level of human involvement required for the trial, and the level of human involvement required for a routine application outside of a RCT setting (discuss under item 21 – generalizability).

	1	2	3	4	5	
subitem not at all important	○	○	○	◉	○	essential

Clear selection

Does your paper address subitem 5-x?

Copy and paste relevant sections from the manuscript (include quotes in quotation marks "like this" to indicate direct quotes from your manuscript), or elaborate on this item by providing additional information not in the ms, or briefly explain why the item is not applicable/relevant for your study

I think this is a very relevant item, although in our study this was a minimal factor: "Throughout the implementation period, the interaction with the users was minimal and based on specific moments of feedback or questions."

5-xi) Report any prompts/reminders used

Report any prompts/reminders used: Clarify if there were prompts (letters, emails, phone calls, SMS) to use the application, what triggered them, frequency etc. It may be necessary to distinguish between the level of prompts/reminders required for the trial, and the level of prompts/reminders for a routine application outside of a RCT setting (discuss under item 21 – generalizability).

	1	2	3	4	5	
subitem not at all important	○	○	○	○	◉	essential

Clear selection

Does your paper address subitem 5-xi? *

Copy and paste relevant sections from the manuscript (include quotes in quotation marks "like this" to indicate direct quotes from your manuscript), or elaborate on this item by providing additional information not in the ms, or briefly explain why the item is not applicable/relevant for your study

No prompts used for this study.

5-xii) Describe any co-interventions (incl. training/support)

Describe any co-interventions (incl. training/support): Clearly state any interventions that are provided in addition to the targeted eHealth intervention, as ehealth intervention may not be designed as stand-alone intervention. This includes training sessions and support [1]. It may be necessary to distinguish between the level of training required for the trial, and the level of training for a routine application outside of a RCT setting (discuss under item 21 – generalizability.

	1	2	3	4	5	
subitem not at all important	○	○	○	◉	○	essential

Clear selection

Does your paper address subitem 5-xii? *

Copy and paste relevant sections from the manuscript (include quotes in quotation marks "like this" to indicate direct quotes from your manuscript), or elaborate on this item by providing additional information not in the ms, or briefly explain why the item is not applicable/relevant for your study

"Before the start of the implementation period, the use of the software was explained through presentation sessions."

6a) Completely defined pre-specified primary and secondary outcome measures, including how and when they were assessed

Does your paper address CONSORT subitem 6a? *

Copy and paste relevant sections from the manuscript (include quotes in quotation marks "like this" to indicate direct quotes from your manuscript), or elaborate on this item by providing additional information not in the ms, or briefly explain why the item is not applicable/relevant for your study

"Questionnaire
Quantitative questionnaire results are reported and summary descriptives are provided. The free text comments were independently reviewed, and themes were extracted by two researchers (Herter and Khuc) to limit any potential sources of error.

Usage data
The present usage analysis extracted the extent of usage based on the number of entered patients, the total number of interactions with the software and the number of completed analyses with the software. The total number of interactions included the user's performed actions such as accessing of patient data or logins. The number of completed analysis refers to the number of patient files that were completed and filled out with the final response regarding the chosen medication. Descriptive statistics were used to estimate the overall usage over the trial period on individual and practice level."

6a-i) Online questionnaires: describe if they were validated for online use and apply CHERRIES items to describe how the questionnaires were designed/deployed

If outcomes were obtained through online questionnaires, describe if they were validated for online use and apply CHERRIES items to describe how the questionnaires were designed/deployed [9].

	1	2	3	4	5	
subitem not at all important	○	○	○	○	◉	essential

Clear selection

Does your paper address subitem 6a-i?

Copy and paste relevant sections from manuscript text

In various ways, also by quotes from above, all relevant CHERRIES checklist items were used in the execution as well as reporting of the research.

"The study protocol was reviewed and determined to meet the requirements for exemption from Ethics Committee (METC) review under the Dutch Medical Research Involving Human Subjects Act (WMO) and to be in accordance with the Dutch Medical Treatment Act (WGBO) and the Dutch Data Protection Act (WBP)."

"Users were invited to complete the questionnaire via email, which directed them to a 9-page web-based survey. They were able to review and change their responses. Each submission was linked to the same username used to access the CDSS. Participation was voluntary, with no incentives provided. Three reminder emails were sent over a two-month period after software usage."

"Thirty out of 34 participating practices submitted at least one response with practices submitting on average 2.23 responses (SD = 1.43). These resulted in 67 healthcare professionals that responded to the questionnaire. All questionnaires were completely filled out. Of these respondents, 31 were assistants and 36 were GPs (Table 1)."

6a-ii) Describe whether and how “use” (including intensity of use/dosage) was defined/measured/monitored

Describe whether and how “use” (including intensity of use/dosage) was defined/measured/monitored (logins, logfile analysis, etc.). Use/adoption metrics are important process outcomes that should be reported in any ehealth trial.

	1	2	3	4	5	
subitem not at all important	○	○	○	◉	○	essential

Clear selection

Does your paper address subitem 6a-ii?

Copy and paste relevant sections from manuscript text

"The present usage analysis extracted the extent of usage based on the number of entered patients, the total number of interactions with the software and the number of completed analyses with the software. The total number of interactions included the user’s performed actions such as accessing of patient data or logins. The number of completed analysis refers to the number of patient files that were completed and filled out with the final response regarding the chosen medication. Descriptive statistics were used to estimate the overall usage over the trial period on individual and practice level."

6a-iii) Describe whether, how, and when qualitative feedback from participants was obtained

Describe whether, how, and when qualitative feedback from participants was obtained (e.g., through emails, feedback forms, interviews, focus groups).

	1	2	3	4	5	
subitem not at all important	○	○	○	◉	○	essential

Clear selection

Does your paper address subitem 6a-iii?

Copy and paste relevant sections from manuscript text

Your answer

6b) Any changes to trial outcomes after the trial commenced, with reasons

Does your paper address CONSORT subitem 6b? *

Copy and paste relevant sections from the manuscript (include quotes in quotation marks "like this" to indicate direct quotes from your manuscript), or elaborate on this item by providing additional information not in the ms, or briefly explain why the item is not applicable/relevant for your study

There were no changes to trial outcomes, not from this survey nor from the article discussing the clinical impact, that was already published (doi: 10.2196/27795).

7a) How sample size was determined

NPT: When applicable, details of whether and how the clustering by care provides or centers was addressed

7a-i) Describe whether and how expected attrition was taken into account when calculating the sample size

Describe whether and how expected attrition was taken into account when calculating the sample size.

	1	2	3	4	5	
subitem not at all important	○	○	○	○	○	essential

Does your paper address subitem 7a-i?

Copy and paste relevant sections from manuscript title (include quotes in quotation marks "like this" to indicate direct quotes from your manuscript), or elaborate on this item by providing additional information not in the ms, or briefly explain why the item is not applicable/relevant for your study

Your answer

7b) When applicable, explanation of any interim analyses and stopping guidelines

Does your paper address CONSORT subitem 7b? *

Copy and paste relevant sections from the manuscript (include quotes in quotation marks "like this" to indicate direct quotes from your manuscript), or elaborate on this item by providing additional information not in the ms, or briefly explain why the item is not applicable/relevant for your study

Also this subitem was not applicable to our research.

8a) Method used to generate the random allocation sequence

NPT: When applicable, how care providers were allocated to each trial group

Does your paper address CONSORT subitem 8a? *

Copy and paste relevant sections from the manuscript (include quotes in quotation marks "like this" to indicate direct quotes from your manuscript), or elaborate on this item by providing additional information not in the ms, or briefly explain why the item is not applicable/relevant for your study

As this study is not an RCT, this has not been done with the same rigor: "The cohort was mostly recruited on caregroup level, and all the practices within a caregroup were obliged to participate."

8b) Type of randomisation; details of any restriction (such as blocking and block size)

Does your paper address CONSORT subitem 8b? *

Copy and paste relevant sections from the manuscript (include quotes in quotation marks "like this" to indicate direct quotes from your manuscript), or elaborate on this item by providing additional information not in the ms, or briefly explain why the item is not applicable/relevant for your study

As this study is not an RCT, this has not been done with the same rigor: "The cohort was mostly recruited on caregroup level, and all the practices within a caregroup were obliged to participate."

9) Mechanism used to implement the random allocation sequence (such as sequentially numbered containers), describing any steps taken to conceal the sequence until interventions were assigned

Does your paper address CONSORT subitem 9? *

Copy and paste relevant sections from the manuscript (include quotes in quotation marks "like this" to indicate direct quotes from your manuscript), or elaborate on this item by providing additional information not in the ms, or briefly explain why the item is not applicable/relevant for your study

As this study is not an RCT, this has not been done with the same rigor: "The cohort was mostly recruited on caregroup level, and all the practices within a caregroup were obliged to participate."

10) Who generated the random allocation sequence, who enrolled participants, and who assigned participants to interventions

Does your paper address CONSORT subitem 10? *

Copy and paste relevant sections from the manuscript (include quotes in quotation marks "like this" to indicate direct quotes from your manuscript), or elaborate on this item by providing additional information not in the ms, or briefly explain why the item is not applicable/relevant for your study

As this study is not an RCT, this has not been done with the same rigor: "The cohort was mostly recruited on caregroup level, and all the practices within a caregroup were obliged to participate."

11a) If done, who was blinded after assignment to interventions (for example, participants, care providers, those assessing outcomes) and how

NPT: Whether or not administering co-interventions were blinded to group assignment

11a-i) Specify who was blinded, and who wasn't

Specify who was blinded, and who wasn't. Usually, in web-based trials it is not possible to blind the participants [1, 3] (this should be clearly acknowledged), but it may be possible to blind outcome assessors, those doing data analysis or those administering co-interventions (if any).

	1	2	3	4	5	
subitem not at all important	○	○	○	◉	○	essential

Clear selection

Does your paper address subitem 11a-i? *

Copy and paste relevant sections from the manuscript (include quotes in quotation marks "like this" to indicate direct quotes from your manuscript), or elaborate on this item by providing additional information not in the ms, or briefly explain why the item is not applicable/relevant for your study

As this study is not an RCT, this has not been done with the same rigor: "The cohort was mostly recruited on caregroup level, and all the practices within a caregroup were obliged to participate."

11a-ii) Discuss e.g., whether participants knew which intervention was the "intervention of interest" and which one was the "comparator"

Informed consent procedures (4a-ii) can create biases and certain expectations - discuss e.g., whether participants knew which intervention was the "intervention of interest" and which one was the "comparator".

	1	2	3	4	5	
subitem not at all important	○	○	○	◉	○	essential

Clear selection

Does your paper address subitem 11a-ii?

Copy and paste relevant sections from the manuscript (include quotes in quotation marks "like this" to indicate direct quotes from your manuscript), or elaborate on this item by providing additional information not in the ms, or briefly explain why the item is not applicable/relevant for your study

As this study is not an RCT, this has not been done with the same rigor: "The cohort was mostly recruited on caregroup level, and all the practices within a caregroup were obliged to participate."

11b) If relevant, description of the similarity of interventions

(this item is usually not relevant for ehealth trials as it refers to similarity of a placebo or sham intervention to a active medication/intervention)

Does your paper address CONSORT subitem 11b? *

Copy and paste relevant sections from the manuscript (include quotes in quotation marks "like this" to indicate direct quotes from your manuscript), or elaborate on this item by providing additional information not in the ms, or briefly explain why the item is not applicable/relevant for your study

As this study is not an RCT, this has not been done with the same rigor: "The cohort was mostly recruited on caregroup level, and all the practices within a caregroup were obliged to participate."

12a) Statistical methods used to compare groups for primary and secondary outcomes

NPT: When applicable, details of whether and how the clustering by care providers or centers was addressed

Does your paper address CONSORT subitem 12a? *

Copy and paste relevant sections from the manuscript (include quotes in quotation marks "like this" to indicate direct quotes from your manuscript), or elaborate on this item by providing additional information not in the ms, or briefly explain why the item is not applicable/relevant for your study

This has been done in the article with regards to the clinical impact (doi: 10.2196/27795).

12a-i) Imputation techniques to deal with attrition / missing values

Imputation techniques to deal with attrition / missing values: Not all participants will use the intervention/comparator as intended and attrition is typically high in ehealth trials. Specify how participants who did not use the application or dropped out from the trial were treated in the statistical analysis (a complete case analysis is strongly discouraged, and simple imputation techniques such as LOCF may also be problematic [4]).

	1	2	3	4	5	
subitem not at all important	○	○	○	◉	○	essential

Clear selection

Does your paper address subitem 12a-i? *

Copy and paste relevant sections from the manuscript (include quotes in quotation marks "like this" to indicate direct quotes from your manuscript), or elaborate on this item by providing additional information not in the ms, or briefly explain why the item is not applicable/relevant for your study

There were no missing values, so this subitem is non applicable to our study.

12b) Methods for additional analyses, such as subgroup analyses and adjusted analyses

Does your paper address CONSORT subitem 12b? *

Copy and paste relevant sections from the manuscript (include quotes in quotation marks "like this" to indicate direct quotes from your manuscript), or elaborate on this item by providing additional information not in the ms, or briefly explain why the item is not applicable/relevant for your study

This has been done in the article with regards to the clinical impact (doi: 10.2196/27795).

X26) REB/IRB Approval and Ethical Considerations [recommended as subheading under "Methods"] (not a CONSORT item)

X26-i) Comment on ethics committee approval

	1	2	3	4	5	
subitem not at all important	○	○	○	◉	○	essential

Clear selection

Does your paper address subitem X26-i?

Copy and paste relevant sections from the manuscript (include quotes in quotation marks "like this" to indicate direct quotes from your manuscript), or elaborate on this item by providing additional information not in the ms, or briefly explain why the item is not applicable/relevant for your study

"The study protocol was reviewed and determined to meet the requirements for exemption from Ethics Committee (METC) review under the Dutch Medical Research Involving Human Subjects Act (WMO) and to be in accordance with the Dutch Medical Treatment Act (WGBO) and the Dutch Data Protection Act (WBP)."

x26-ii) Outline informed consent procedures

Outline informed consent procedures e.g., if consent was obtained offline or online (how? Checkbox, etc.?), and what information was provided (see 4a-ii). See [6] for some items to be included in informed consent documents.

	1	2	3	4	5	
subitem not at all important	○	○	○	○	○	essential

Does your paper address subitem X26-ii?

Copy and paste relevant sections from the manuscript (include quotes in quotation marks "like this" to indicate direct quotes from your manuscript), or elaborate on this item by providing additional information not in the ms, or briefly explain why the item is not applicable/relevant for your study

Your answer

X26-iii) Safety and security procedures

Safety and security procedures, incl. privacy considerations, and any steps taken to reduce the likelihood or detection of harm (e.g., education and training, availability of a hotline)

	1	2	3	4	5	
subitem not at all important	○	○	○	○	○	essential

Does your paper address subitem X26-iii?

Copy and paste relevant sections from the manuscript (include quotes in quotation marks "like this" to indicate direct quotes from your manuscript), or elaborate on this item by providing additional information not in the ms, or briefly explain why the item is not applicable/relevant for your study

Your answer

RESULTS

13a) For each group, the numbers of participants who were randomly assigned, received intended treatment, and were analysed for the primary outcome

NPT: The number of care providers or centers performing the intervention in each group and the number of patients treated by each care provider in each center

Does your paper address CONSORT subitem 13a? *

Copy and paste relevant sections from the manuscript (include quotes in quotation marks "like this" to indicate direct quotes from your manuscript), or elaborate on this item by providing additional information not in the ms, or briefly explain why the item is not applicable/relevant for your study

This paper is not an RCT and this subitem is non applicable.

13b) For each group, losses and exclusions after randomisation, together with reasons

Does your paper address CONSORT subitem 13b? (NOTE: Preferably, this is shown in a CONSORT flow diagram) *

Copy and paste relevant sections from the manuscript (include quotes in quotation marks "like this" to indicate direct quotes from your manuscript), or elaborate on this item by providing additional information not in the ms, or briefly explain why the item is not applicable/relevant for your study

This paper is not an RCT and this subitem is non applicable.

13b-i) Attrition diagram

Strongly recommended: An attrition diagram (e.g., proportion of participants still logging in or using the intervention/comparator in each group plotted over time, similar to a survival curve) or other figures or tables demonstrating usage/dose/engagement.

	1	2	3	4	5	
subitem not at all important	○	○	○	●	○	essential

Clear selection

Does your paper address subitem 13b-i?

Copy and paste relevant sections from the manuscript or cite the figure number if applicable (include quotes in quotation marks "like this" to indicate direct quotes from your manuscript), or elaborate on this item by providing additional information not in the ms, or briefly explain why the item is not applicable/relevant for your study

"Of the original 36 practices, 34 started with the implementation study (95%). 31 practices continued using the tool throughout the pilot period, with an 9% dropout in the first 8 weeks."

14a) Dates defining the periods of recruitment and follow-up

Does your paper address CONSORT subitem 14a? *

Copy and paste relevant sections from the manuscript (include quotes in quotation marks "like this" to indicate direct quotes from your manuscript), or elaborate on this item by providing additional information not in the ms, or briefly explain why the item is not applicable/relevant for your study

""Users were invited to complete the questionnaire via email, which directed them to a 9-page web-based survey. They were able to review and change their responses. Each submission was linked to the same username used to access the CDSS. Participation was voluntary, with no incentives provided. Three reminder emails were sent over a two-month period after software usage."

14a-i) Indicate if critical “secular events” fell into the study period

Indicate if critical “secular events” fell into the study period, e.g., significant changes in Internet resources available or “changes in computer hardware or Internet delivery resources”

	1	2	3	4	5	
subitem not at all important	○	○	○	◉	○	essential

Clear selection

Does your paper address subitem 14a-i?

Copy and paste relevant sections from the manuscript (include quotes in quotation marks "like this" to indicate direct quotes from your manuscript), or elaborate on this item by providing additional information not in the ms, or briefly explain why the item is not applicable/relevant for your study

Your answer

14b) Why the trial ended or was stopped (early)

Does your paper address CONSORT subitem 14b? *

Copy and paste relevant sections from the manuscript (include quotes in quotation marks "like this" to indicate direct quotes from your manuscript), or elaborate on this item by providing additional information not in the ms, or briefly explain why the item is not applicable/relevant for your study

"To evaluate the usability and perceived usefulness of the CDSS, an implementation study was conducted in 36 primary care practices for a period of four months." - this period was determined in advance.

15) A table showing baseline demographic and clinical characteristics for each group

NPT: When applicable, a description of care providers (case volume, qualification, expertise, etc.) and centers (volume) in each group

?

Does your paper address CONSORT subitem 15? *

Copy and paste relevant sections from the manuscript (include quotes in quotation marks "like this" to indicate direct quotes from your manuscript), or elaborate on this item by providing additional information not in the ms, or briefly explain why the item is not applicable/relevant for your study

"Table 1. Characteristics of GPs and assistants' questionnaire respondents relative to registered Dutch GPs."

15-i) Report demographics associated with digital divide issues

In ehealth trials it is particularly important to report demographics associated with digital divide issues, such as age, education, gender, social-economic status, computer/Internet/ehealth literacy of the participants, if known.

	1	2	3	4	5	
subitem not at all important	○	○	○	●	○	essential

Clear selection

Does your paper address subitem 15-i? *

Copy and paste relevant sections from the manuscript (include quotes in quotation marks "like this" to indicate direct quotes from your manuscript), or elaborate on this item by providing additional information not in the ms, or briefly explain why the item is not applicable/relevant for your study

"Table 1. Characteristics of GPs and assistants' questionnaire respondents relative to registered Dutch GPs." - and general attitudes with regards to (data-based) innovation in healthcare.

16) For each group, number of participants (denominator) included in each analysis and whether the analysis was by original assigned groups

16-i) Report multiple “denominators” and provide definitions

Report multiple “denominators” and provide definitions: Report N’s (and effect sizes) “across a range of study participation [and use] thresholds” [1], e.g., N exposed, N consented, N used more than x times, N used more than y weeks, N participants “used” the intervention/comparator at specific pre-defined time points of interest (in absolute and relative numbers per group). Always clearly define “use” of the intervention.

	1	2	3	4	5	
subitem not at all important	○	○	○	○	◉	essential

Clear selection

Does your paper address subitem 16-i? *

Copy and paste relevant sections from the manuscript (include quotes in quotation marks "like this" to indicate direct quotes from your manuscript), or elaborate on this item by providing additional information not in the ms, or briefly explain why the item is not applicable/relevant for your study

This is non applicable since this study is not an RCT.

16-ii) Primary analysis should be intent-to-treat

Primary analysis should be intent-to-treat, secondary analyses could include comparing only “users”, with the appropriate caveats that this is no longer a randomized sample (see 18-i).

	1	2	3	4	5	
subitem not at all important	○	○	○	◉	○	essential

Clear selection

Does your paper address subitem 16-ii?

Copy and paste relevant sections from the manuscript (include quotes in quotation marks "like this" to indicate direct quotes from your manuscript), or elaborate on this item by providing additional information not in the ms, or briefly explain why the item is not applicable/relevant for your study

The "Evaluation Outcomes" is the primary section here.

17a) For each primary and secondary outcome, results for each group, and the estimated effect size and its precision (such as 95% confidence interval)

Does your paper address CONSORT subitem 17a? *

Copy and paste relevant sections from the manuscript (include quotes in quotation marks "like this" to indicate direct quotes from your manuscript), or elaborate on this item by providing additional information not in the ms, or briefly explain why the item is not applicable/relevant for your study

Since this is an descriptive study making use of ordinal Likert scale data with a relatively small sample size, this has not been included at this stage.

17a-i) Presentation of process outcomes such as metrics of use and intensity of use

In addition to primary/secondary (clinical) outcomes, the presentation of process outcomes such as metrics of use and intensity of use (dose, exposure) and their operational definitions is critical. This does not only refer to metrics of attrition (13-b) (often a binary variable), but also to more continuous exposure metrics such as "average session length". These must be accompanied by a technical description how a metric like a "session" is defined (e.g., timeout after idle time) [1] (report under item 6a).

	1	2	3	4	5	
subitem not at all important	○	○	○	○	○	essential

Does your paper address subitem 17a-i?

Copy and paste relevant sections from the manuscript (include quotes in quotation marks "like this" to indicate direct quotes from your manuscript), or elaborate on this item by providing additional information not in the ms, or briefly explain why the item is not applicable/relevant for your study

Your answer

17b) For binary outcomes, presentation of both absolute and relative effect sizes is recommended

Does your paper address CONSORT subitem 17b? *

Copy and paste relevant sections from the manuscript (include quotes in quotation marks "like this" to indicate direct quotes from your manuscript), or elaborate on this item by providing additional information not in the ms, or briefly explain why the item is not applicable/relevant for your study

Non applicable for our research.

18) Results of any other analyses performed, including subgroup analyses and adjusted analyses, distinguishing pre-specified from exploratory

Does your paper address CONSORT subitem 18? *

Copy and paste relevant sections from the manuscript (include quotes in quotation marks "like this" to indicate direct quotes from your manuscript), or elaborate on this item by providing additional information not in the ms, or briefly explain why the item is not applicable/relevant for your study

Non applicable for our research.

18-i) Subgroup analysis of comparing only users

A subgroup analysis of comparing only users is not uncommon in ehealth trials, but if done, it must be stressed that this is a self-selected sample and no longer an unbiased sample from a randomized trial (see 16-iii).

	1	2	3	4	5	
subitem not at all important	○	○	○	◉	○	essential

Clear selection

Does your paper address subitem 18-i?

Copy and paste relevant sections from the manuscript (include quotes in quotation marks "like this" to indicate direct quotes from your manuscript), or elaborate on this item by providing additional information not in the ms, or briefly explain why the item is not applicable/relevant for your study

Non applicable for our research, the potential bias of our research sample is discussed at various points in the article though.

19) All important harms or unintended effects in each group
(for specific guidance see CONSORT for harms)

Does your paper address CONSORT subitem 19? *

Copy and paste relevant sections from the manuscript (include quotes in quotation marks "like this" to indicate direct quotes from your manuscript), or elaborate on this item by providing additional information not in the ms, or briefly explain why the item is not applicable/relevant for your study

Non applicable for our research.

19-i) Include privacy breaches, technical problems

Include privacy breaches, technical problems. This does not only include physical "harm" to participants, but also incidents such as perceived or real privacy breaches [1], technical problems, and other unexpected/unintended incidents. "Unintended effects" also includes unintended positive effects [2].

	1	2	3	4	5	
subitem not at all important	○	○	○	○	○	essential

Does your paper address subitem 19-i?

Copy and paste relevant sections from the manuscript (include quotes in quotation marks "like this" to indicate direct quotes from your manuscript), or elaborate on this item by providing additional information not in the ms, or briefly explain why the item is not applicable/relevant for your study

Your answer

19-ii) Include qualitative feedback from participants or observations from staff/researchers

Include qualitative feedback from participants or observations from staff/researchers, if available, on strengths and shortcomings of the application, especially if they point to unintended/unexpected effects or uses. This includes (if available) reasons for why people did or did not use the application as intended by the developers.

	1	2	3	4	5	
subitem not at all important	○	○	○	○	○	essential

Does your paper address subitem 19-ii?

Copy and paste relevant sections from the manuscript (include quotes in quotation marks "like this" to indicate direct quotes from your manuscript), or elaborate on this item by providing additional information not in the ms, or briefly explain why the item is not applicable/relevant for your study

"There were four free text fields in the questionnaire that allowed users to further elaborate on some of their responses. The first question requested users to further elaborate on how the software was integrated into their daily activities. The main theme that emerged was that the use of the software required too much additional work and was too time consuming. A second theme that emerged was regarding how the tool was used. It was stated that it was mostly assistants who interacted with the software by entering the patients. The presented information was then used by the GP. This also resulted in redistribution of work and extra discussion between doctor and assistant.

The second question, concerning the users' main intentions to use the system, was answered by 70% of respondents. The most frequently stated theme was the opportunity of added informative value that the user's saw in decision support systems. The second theme that emerged was the potential of improved care by delivery of more personalized treatments.

The final question requested further suggestions to improve the software, with the most common suggestions related to a request to integrate the tool into the EHR systems. Lastly, respondents suggested the added value of additional training on how the software should be used well."

DISCUSSION

22) Interpretation consistent with results, balancing benefits and harms, and considering other relevant evidence

NPT: In addition, take into account the choice of the comparator, lack of or partial blinding, and unequal expertise of care providers or centers in each group

22-i) Restate study questions and summarize the answers suggested by the data, starting with primary outcomes and process outcomes (use)

Restate study questions and summarize the answers suggested by the data, starting with primary outcomes and process outcomes (use).

	1	2	3	4	5	
subitem not at all important	○	○	○	○	◉	essential

Clear selection

Does your paper address subitem 22-i? *

Copy and paste relevant sections from the manuscript (include quotes in quotation marks "like this" to indicate direct quotes from your manuscript), or elaborate on this item by providing additional information not in the ms, or briefly explain why the item is not applicable/relevant for your study

We have followed the JMIR structure of discussing our results, and this subitem is addressed.

22-ii) Highlight unanswered new questions, suggest future research

Highlight unanswered new questions, suggest future research.

	1	2	3	4	5	
subitem not at all important	○	○	○	◉	○	essential

Clear selection

Does your paper address subitem 22-ii?

Copy and paste relevant sections from the manuscript (include quotes in quotation marks "like this" to indicate direct quotes from your manuscript), or elaborate on this item by providing additional information not in the ms, or briefly explain why the item is not applicable/relevant for your study

"The use of the survey enables us to obtain relatively unbiased results from a large number of users. In the construction of the questionnaire the UTUAT model was used, allowing to structure the results and additional topics of specific interest were added. However, our particular survey was not fully validated before conduction. Doing an extensive pilot with a group of GPs that were not part of the study but were familiar with the software, helped in assuring that the questions were interpreted and understood well. To further guarantee the reliability, validity and quality of the survey items, SQP was used.

Another point of attention of the research is that the assistants had not been involved in the design phase of the CDSS, whereas they played an important role in the treatment of the UTIs in practice. In many participating practices, much of the work was done by assistants. As such their role in the care path should not have been underestimated and future design should ensure that all potential user groups are included from the beginning. The different uses of the tool for assistants and GPs led to a redistribution of work, which resulted in less work. Nevertheless, evaluating and comparing the results between GP and assistant did not yield significant differences."

20) Trial limitations, addressing sources of potential bias, imprecision, and, if relevant, multiplicity of analyses

20-i) Typical limitations in ehealth trials

Typical limitations in ehealth trials: Participants in ehealth trials are rarely blinded. Ehealth trials often look at a multiplicity of outcomes, increasing risk for a Type I error. Discuss biases due to non-use of the intervention/usability issues, biases through informed consent procedures, unexpected events.

	1	2	3	4	5	
subitem not at all important	○	○	○	◉	○	essential

Clear selection

Does your paper address subitem 20-i? *

Copy and paste relevant sections from the manuscript (include quotes in quotation marks "like this" to indicate direct quotes from your manuscript), or elaborate on this item by providing additional information not in the ms, or briefly explain why the item is not applicable/relevant for your study

"In general, participants stated to have a positive attitude towards change in healthcare. Most of the participants also stated that they would like to increase the amount of evidence in their daily practice, and that they see an opportunity in the use of observational data doing so. Next to that the participants indicated that the NHG guideline leads to better patient care, and the participants mostly stated that the NHG guideline is based on sufficient evidence. This gives us reason to conclude that the participant sample was relatively positive towards change and several ways of increasing evidence in their daily practice."

21) Generalisability (external validity, applicability) of the trial findings

NPT: External validity of the trial findings according to the intervention, comparators, patients, and care providers or centers involved in the trial

21-i) Generalizability to other populations

Generalizability to other populations: In particular, discuss generalizability to a general Internet population, outside of a RCT setting, and general patient population, including applicability of the study results for other organizations

	1	2	3	4	5	
subitem not at all important	○	○	○	○	○	essential

Does your paper address subitem 21-i?

Copy and paste relevant sections from the manuscript (include quotes in quotation marks "like this" to indicate direct quotes from your manuscript), or elaborate on this item by providing additional information not in the ms, or briefly explain why the item is not applicable/relevant for your study

Your answer

21-ii) Discuss if there were elements in the RCT that would be different in a routine application setting

Discuss if there were elements in the RCT that would be different in a routine application setting (e.g., prompts/reminders, more human involvement, training sessions or other co-interventions) and what impact the omission of these elements could have on use, adoption, or outcomes if the intervention is applied outside of a RCT setting.

	1	2	3	4	5	
subitem not at all important	○	○	○	○	○	essential

Does your paper address subitem 21-ii?

Copy and paste relevant sections from the manuscript (include quotes in quotation marks "like this" to indicate direct quotes from your manuscript), or elaborate on this item by providing additional information not in the ms, or briefly explain why the item is not applicable/relevant for your study

Your answer

OTHER INFORMATION

23) Registration number and name of trial registry

Does your paper address CONSORT subitem 23? *

Copy and paste relevant sections from the manuscript (include quotes in quotation marks "like this" to indicate direct quotes from your manuscript), or elaborate on this item by providing additional information not in the ms, or briefly explain why the item is not applicable/relevant for your study

Trial Registration: ClinicalTrials.gov NCT04408976.

24) Where the full trial protocol can be accessed, if available

Does your paper address CONSORT subitem 24? *

Cite a Multimedia Appendix, other reference, or copy and paste relevant sections from the manuscript (include quotes in quotation marks "like this" to indicate direct quotes from your manuscript), or elaborate on this item by providing additional information not in the ms, or briefly explain why the item is not applicable/relevant for your study

The full trial protocol can be accessed upon request.

25) Sources of funding and other support (such as supply of drugs), role of funders

Does your paper address CONSORT subitem 25? *

Copy and paste relevant sections from the manuscript (include quotes in quotation marks "like this" to indicate direct quotes from your manuscript), or elaborate on this item by providing additional information not in the ms, or briefly explain why the item is not applicable/relevant for your study

"Health insurers funded the research partly."

X27) Conflicts of Interest (not a CONSORT item)

X27-i) State the relation of the study team towards the system being evaluated

In addition to the usual declaration of interests (financial or otherwise), also state the relation of the study team towards the system being evaluated, i.e., state if the authors/evaluators are distinct from or identical with the developers/sponsors of the intervention.

	1	2	3	4	5	
subitem not at all important	○	○	○	●	○	essential

Clear selection

Does your paper address subitem X27-i?

Copy and paste relevant sections from the manuscript (include quotes in quotation marks "like this" to indicate direct quotes from your manuscript), or elaborate on this item by providing additional information not in the ms, or briefly explain why the item is not applicable/relevant for your study

"Herter is a PhD candidate at Leiden University Medical Center (LUMC) as well as a former director at Pacmed. Khuc worked at Pacmed during their contribution to the research and paper."

About the CONSORT EHEALTH checklist

As a result of using this checklist, did you make changes in your manuscript? *

- ○ yes, major changes
- ◉ yes, minor changes
- ○ no

What were the most important changes you made as a result of using this checklist?

Please see above, mostly the CHERRIES checklist and additions to the Methods section.

How much time did you spend on going through the checklist INCLUDING making changes in your manuscript *

Multiple hours, probably half a day.

As a result of using this checklist, do you think your manuscript has improved? *

- (selected) yes
- no
- Other:

Would you like to become involved in the CONSORT EHEALTH group?

This would involve for example becoming involved in participating in a workshop and writing an "Explanation and Elaboration" document

- yes
- no
- (selected) Other: Depends on the request.

Clear selection

Any other comments or questions on CONSORT EHEALTH

Your answer

STOP - Save this form as PDF before you click submit

To generate a record that you filled in this form, we recommend to generate a PDF of this page (on a Mac, simply select "print" and then select "print as PDF") before you submit it.

When you submit your (revised) paper to JMIR, please upload the PDF as supplementary file.

Don't worry if some text in the textboxes is cut off, as we still have the complete information in our database. Thank you!

Final step: Click submit !

Click submit so we have your answers in our database!

Submit   Clear form

Never submit passwords through Google Forms.

This content is neither created nor endorsed by Google. - Contact form owner - Terms of Service - Privacy Policy

Does this form look suspicious? Report

Google Forms
